# High‐resolution genome and genetic map of tetraploid *Allium porrum* expose pericentromeric recombination

**DOI:** 10.1002/tpg2.70159

**Published:** 2025-12-08

**Authors:** Ronald Nieuwenhuis, Roeland Voorrips, Danny Esselink, Thamara Hesselink, Elio Schijlen, Paul Arens, Jan Cordewener, Hetty C. van den Broeck, Olga Scholten, Sander Peters

**Affiliations:** ^1^ Business Unit of Bioscience, Cluster Applied Bioinformatics Wageningen University and Research Wageningen The Netherlands; ^2^ Business Unit of Plant Breeding Wageningen University and Research Wageningen The Netherlands

## Abstract

We present the first reference genome of the highly heterozygous autotetraploid *Allium porrum* (leek). Combining long‐read sequencing with single‐nucleotide polymorphism (SNP)‐array screening of two experimental F1 populations, we generated a genetic map with 11,429 SNP markers across eight linkage groups and a chromosome‐scale assembly of *A. porrum* (leek) totaling 15.2 Gbp in size. The high quality of the reference genome is substantiated by 97.2% BUSCO completeness and a mapping rate of 96% for full‐length transcripts. The linkage map exposes the recombination landscape of leek and confirms that crossovers are predominantly proximal, located to the centromeres, contrasting with distal recombination landscapes observed in other *Allium* species. Comparative genomics reveals structural rearrangements between *A. porrum* and its relatives (*Allium fistulosum*, *Allium sativum*, and *Allium cepa*), suggesting a closer genomic relationship to *A. sativum*. Our annotated high‐quality reference genome delivers crucial insights into the leek genome structure, recombination landscape, and evolutionary relationships within the *Allium* genus, with implications for species compatibility in breeding programs, facilitating marker‐assisted selection and genetic improvement in leek.

## Plain Language Summary

Leek is an important vegetable crop, but its very large and complex genome had not been fully mapped until now. Understanding its genome structure can help researchers improve breeding methods and better use related species. In this study, we produced the first chromosome‐scale reference genome of leek, which has four copies of each chromosome (autotetraploid) and spans over 15 billion DNA letters. We also examined how genetic material is exchanged during reproduction and found that, unlike in related species, most recombination in leek occurs near the centers of chromosomes. By comparing the leek genome to those of its relatives, such as onion and garlic, we identified key structural differences and found that leek is more closely related to garlic. This new reference genome offers valuable insights into leek evolution and genome organization. It provides a powerful resource for marker‐assisted selection and modern breeding strategies aimed at improving leek varieties.

**Abbreviations**
CO: crossover
HiFi: high fidelity
HMW: high molecular weight
LOD: log of odds
SNP: single‐nucleotide polymorphism

## 1 INTRODUCTION

Leek (*Allium porrum* syn. *Allium ampeloprasum* var. *porrum*) is a member of the *Allium* genus to which also the well‐known onion (*Allium cepa*), shallot (*A. cepa* var. *aggregatum*), garlic (*Allium sativum*), Japanese bunching onion or Welsh onion (*Allium fistulosum*), scallion (*A. cepa* var. *cepa*), chives (*Allium schoenoprasum*), and Chinese onion (*Allium chinense*) vegetables belong. Historically, *Allium* species have been cultivated and appreciated for consumption already from the second millennium BC onward, as evidenced by dried specimens found from archeological sites in Egypt and Mesopotamia (Hehn, 1870; Zohary et al., 2012) and documented Roman recipes (Sanderson & Renfrew, 2005). Today, cultivated leek is globally appreciated for its mild onion‐like taste and nutritional value, with high content of vitamin B6, C and K, folate, iron, and manganese.

Leek is a cross‐fertilizing species and is generally considered a tetraploid (2*n* = 4*x* = 32) (Kik et al., 2021). Both auto‐ and (weak) segmental allopolyploidy are reported in different studies based on varying levels of multivalent formation (Kadry & Kamel, 1955; Koul & Gohil, 1970; Levan, 1940; Murín, 1964). Although leek can self‐fertilize to a limited extent of 20%, it displays strong inbreeding depression with plant‐weight reductions of up to 60% (Berninger & Buret, 1967). In general, modern leek breeding concentrates on better crop uniformity, higher yield, and pest and disease resistance. To obtain better uniformity and higher yield, mostly F1 hybrids are exploited as commercial varieties. Because of intensive selection by leek growers since the last century, a significant amount of genetic variation originally present in the old landraces has been lost in leek germplasm. This genetic erosion has further challenged leek breeders in the identification and introgression of resistance to leek moth (*Acrolepiopsis assectella*), leek fly (*Delia antiqua*), thrips (*Thrips tabaci*), onion leaf miner (*Liriomyza cepae*), stem and bulb nematodes (*Ditylenchus dipsaci*, *Meloidogyne* spp., *Pratylenchus penetrans*, *Paratrichodorus* spp., *Trichodorus* spp., and *Longidorus* spp.), smudge (*Colletotrichum circinans*), leaf blotch (*Cladosporium allii*), white tip disease (*Phytophthora porri*), purple blotch (*Alternaria porri*), leek rust (*Puccinia allii*), basal rot (*Fusarium culmorum*), white rot (*Sclerotium cepivorum*), black mould (*Pleospora herbarum*), bacterial diseases caused by *Pseudomonas* spp. and *Erwinia* spp., and aphid transmitted viral diseases such as Leek Yellow Stripe Virus (Becue, 2003; Lorbeer et al., 2002; Mark et al., 2002; Salomon, 2002). Until today, resistance to many of these diseases and pests in cultivated leek crops is absent.

For genetic improvement of Allium crops, crop wild relatives (CWR) are considered important genetic resources (Kik et al., 2021). Despite the successful use of introgression breeding with cross‐fertilizing wild species to transfer beneficial alleles in many target crops, Allium breeders have faced significant challenges in using wide hybridization for leek crop improvement. The success of introgression breeding relies on meiotic recombination, a biological process during which homoeologous chromosomes exchange segments through crossovers (COs), enabling the transfer of donor alleles into the recipient genome. As observed for many organisms, also in Allium species, COs are not randomly distributed, and their distribution and localization appear genetically and strictly controlled (Kudryavtseva et al., 2023). Furthermore, the designation of COs varies considerably between *Allium* species. Recently, Kudryavtseva et al. (2023) demonstrated that *A. fistulosum* COs predominantly occur in the proximal chromosome regions, while in *A. cepa*, COs mainly manifest in the distal and interstitial chromosome regions. These differences may result in homoeologous chromosome pairing disorders during meiosis in hybrids, leading to reduced COs in regions containing beneficial alleles and hampering introgression breeding. Indeed, Allium introgression lines often display genome instability through a phenomenon known as genome dominance, manifested by unequal inheritance and loss of introgressed genetic donor material in successive generations, further impeding the employment of wide hybridization in practical Allium breeding (Kopecký et al., 2022). Thus, understanding of the recombination and CO landscapes is crucial for successful introgression breeding in Allium crops.

The development of a reference genome and genetic map offers a significant opportunity to overcome many of these challenges in leek breeding. These tools enable the assessment of genetic variation and inheritance, facilitating more efficient breeding strategies. A major advantage is the improved ability to identify beneficial haplotypes for disease resistance. Given the leek's widespread susceptibility to pests, fungi, bacteria, and viruses, distinguishing between parental haplotypes in an outcrossing autotetraploid would allow breeders to track, select, and monitor the retention of specific resistance alleles over generations. Additionally, linkage maps help clarify recombination patterns and CO distribution. Since COs are not uniformly distributed, a genome assembly and genetic map provide insight into CO patterns, allowing breeders to select compatible breeding lines for successful introgression breeding. This is particularly helpful when incorporating alleles from CWR and avoiding homoeologous chromosome pairing issues (Jones et al., 1996; Khazanehdari & Jones, 1997; Kiełkowska, 2012). Linkage maps also help identify and track deleterious recessive alleles that may negatively affect plant fitness, aiding breeders in selecting complementary parental lines with optimized agronomic performance and disease resistance, while minimizing inbreeding depression. Structural variation detection is another relevant use of reference genomes and genetic maps. Large‐scale genomic variations, such as copy number variations, inversions, and translocations, can influence key agronomic traits. By combining detailed genome sequences with genetic map data, breeders can better understand these variations and selectively retain or eliminate structural variants that impact crop performance. Genetic maps also play a crucial role in stabilizing introgression lines, allowing the effective transfer of beneficial traits by tracking wild allele inheritance and identifying regions prone to instability.

The extensive genome size, as reported by Arumuganathan and Earle (1991) and indicated by *C*‐values (https://cvalues.science.kew.org/), the autotetraploid heterozygous nature, and the highly repetitive structure of the leek genome have long posed challenges for genome assembly and genetic map construction. In this paper, we present the construction of the first chromosome‐scale reference genome for leek, along with the development of a genetic map, marking a significant milestone in Allium genomics and breeding. By resolving the complexities of this genome at a chromosome scale, this study provides a foundation for dissecting genetic variation, understanding recombination landscapes, and accelerating breeding efforts. Ultimately, this achievement paves the way for more resilient, genetically diverse, and high‐performing leek cultivars, ensuring the sustainable advancement of this globally important crop.

## 2 MATERIALS AND METHODS

### 2.1 Plant material

We established a genome sequence from an individual leek plant of accession Leidse prei 2018‐94, a landrace/grower selection from the Netherlands. Linkage mapping was performed in two populations: one F1 of the cross 34012‐7 (a genic male sterile [gms] plant donated by Bejo Zaden, the Netherlands) × the Leidse prei 2018‐94 plant mentioned above, consisting of 187 plants, and one F1 of the cross 17169‐29 (a gms plant donated by Nunhems/BASF Crop Science, the Netherlands) × *A. porrum*, consisting of 248 plants.

### 2.2 DNA isolation and library prep

Seven clones of the leek father plant Leidse prei 2018‐94 were used for tissue harvest. Multiple young most inner leaves were harvested, snap frozen in liquid nitrogen, and stored at −80°C. Approximately 1.5 g of leaf material was ground with liquid nitrogen and used for high molecular weight (HMW) gDNA isolation using the NucleoBond HMW DNA kit following the manufacturer's instructions (Machery‐Nagel). Additionally, 1 g of leaf material was ground with liquid nitrogen and used for HMW gDNA isolation using the MagMAX Plant DNA kit following the manufacturer's instructions (Applied Biosystems). DNA quantity, purity, and fragment size were determined using Qubit (Invitrogen), OD (optical density) values (NanoDrop), and Fragment Analyzer (Agilent). The obtained gDNA was sheared in different rounds by Megaruptor 2 (Diagenode) with a 20–30 kbp target fragment size. Small fragments were removed by utilizing Blue Pippin (Sage Science) for size selection. Size‐selected DNA was used for six SMRTbell library preps using SMRTbell Express Template Preparation kit 2.0 according to the manufacturer's instructions (Pacbio; Procedure‐Checklist‐Preparing‐HiFi‐SMRTbell‐Libraries‐using‐SMRTbell‐Express‐Template‐Prep, where HiFi stands for high fidelity). SMRTbell libraries were subjected to DNA polymerase complexing using Sequel binding kit v2.0/3.0 and Sequel Polymerase 3.0/2.2. Final sequencing was done using sequence primer v4/v5 on a Pacbio Sequel‐II/Sequel‐IIe instrument (see Section 2.3). Sequencing reactions were performed with sequencing kit 2.0/3.0, using adaptive loading, 65–85 pM on plate loading concentration, 120 or 240 min immobilization time, and 30 h movie time per SMRT cell.

### 2.3 Genome sequencing

Long insert libraries of 15 and 19 kbp were sequenced on the PacBio Sequel‐II(e) platform using 44 SMRT cells (Table S1). Subsequent circular consensus sequences (CCS) were called using the pbccs v5.0.0 command line utility. HiFi reads were defined as CCS reads having a minimum number of three passes and a mean read quality score of Q20. Quality control per SMRTcell was checked via SMRTlink and an in‐house pipeline including FastQC v0.11.9 (Andrews, 2010), KMC v3.1.1 (Kokot et al., 2017), Smudgeplot v0.2.3dev_rn (Ranallo‐Benavidez et al., 2020), GenomeScope v2 (Ranallo‐Benavidez et al., 2020), and BlastN v2.11.0+ (Altschul et al., 1990; Camacho et al., 2009) with the NCBI nt, plastid, and mitochondrion publicly available databases downloaded April 2, 2021 (Sayers et al., 2024). Reads produced from different libraries were then combined into a single dataset for further analyses.

### 2.4 Contig assembly

Combined reads of all sequenced libraries were assembled using hifiasm v0.15.1‐r334 (Cheng et al., 2021) with output settings for primary and alternative assembly selected. All contigs from both the primary and alternative assemblies were screened for contamination using NCBI Foreign Contamination Screen v0.4.0, and nonsubject sequences were subsequently removed (Astashyn et al., 2024). Splitting of contigs was done with NCBI Adaptor v0.4.0 in case of detected adaptor sequences. Any remaining adaptors sequences were removed to avoid assembly errors (Figure S1). BUSCO v5.2.2 (Manni, Berkeley, Seppey, Simão, et al., 2021; Manni, Berkeley, Seppey, & Zdobnov, 2021; Simão et al., 2015) was used with the embryophyte odb10 database (September 10, 2020) to check the completeness of conserved single‐copy orthologs. Further purging of the primary assembly was performed in a customized manner to accommodate the large dataset and limited sequence coverage. This approach optimized single‐copy BUSCO gene percentage in a minimal set of contigs (Figure S2).

Selected contigs were used for subsequent annotation and scaffolding. *k*‐mer‐based completeness checks were omitted due to the heterozygous and autopolyploid nature of the sequenced specimen, with the purged result representing only a pseudo‐haploid version of the genome.

Reference‐based assembly was applied to the leek WGS HiFi dataset to assemble the mitochondrion and chloroplast genomes. For the plastid genome, ptGAUL v1.0.5 (Zhou et al., 2023) was used with the *A. ampeloprasum* reference sequence (NC_044666.1). For the mitochondrial genome, mitoHIFI v3.0.1 (Uliano‐Silva et al., 2023) and ptGAUL v1.0.5 assemblers were used with references from *A. cepa* and *Allium sativa*.

### 2.5 RNA isolation and library prep

For RNA isolation, material of different tissues (green leaf, green/white stem section, basal plate section, and roots) from the leek father plant (2018‐94‐02) was collected. In addition, flower tissue was collected from a different plant (leek mother plant 17169‐29 gms), immediately snap frozen in liquid nitrogen, and stored at −80°C. RNA from the father plant tissues was isolated using the Ambion PureLinkRNA Mini Kit (Life Technologies). RNA from the mother plant flower tissue was isolated using the ZymoBIOMICS RNA Mini prep Kit (Zymo Research). Subsequently, RNA quantity and purity were analyzed by Qubit (Invitrogen), OD values (NanoDrop), and Bioanalyzer RNA plant pico assay (Agilent).

Of each tissue, 300 ng total RNA was used to create a barcoded IsoSeq library following the manufacturer's guidelines (Pacbio; Procedure‐Checklist‐Iso‐Seq‐Express‐Template‐Preparation‐for‐Sequel‐and‐Sequel‐II‐Systems). SMRTbell library yield was quantified by Qubit (Invitrogen), and SMRTbell sizes were checked by Bioanalyzer High Sensitivity DNA Assay (Agilent). Libraries were pooled equimolar, subjected to DNA Polymerase SMRTbell complexing using Sequel II Binding kit 2.0. and primer v4 prior to loading on 4 SMRT cells with 50–58 pM on plate loading concentration. Sequencing reaction was performed on a PacBio Sequel‐II system with 24‐h movie time.

### 2.6 Transcriptome sequencing

Genetic diversity was mined for array probe design by using RNA‐seq of 14 *A. porrum* accessions including the mapping population parents Leidse prei 2018‐94, MS34012‐7, and the species *Allium lusitanicum* and *Allium eduardii* (Table S2). RNA‐seq libraries of young plantlets and leaf tissue were sequenced on an Illumina Novaseq6000 platform using an S2 flow cell. Base calling and initial quality filtering of raw sequencing data were done with bcl2fastq v2.20.0.422 (https://emea.support.illumina.com/downloads/bcl2fastq‐conversion‐software‐v2‐20.html) using default settings.

Next, full‐length transcripts from different tissues of the sequenced subject were sequenced, using PacBio IsoSeq on a Sequel‐IIe platform for annotation of the de novo assembled genome. Consensus reads were called with the ccs v5.0.0 command‐line utility of PacBio. HiFi reads were generated using the same specifications as used for the genomic reads. Reads were then stripped of primers and demultiplexed using lima v2.0.0 (https://github.com/PacificBiosciences/barcoding). Poly‐A tails were trimmed, and concatemers were removed using isoseq3 v3.4.0 (https://github.com/PacificBiosciences/IsoSeq) to generate full‐length non‐concatemer reads, which were subsequently clustered using isoseq3 cluster (https://github.com/ylipacbio/IsoSeq3) without final polishing.

#### 2.6.1 Axiom array design

To create a genetic map, first, a reference transcriptome sequence set was constructed by clustering all the IsoSeq data of Leidse prei 2018‐94 with isONclust (Sahlin & Medvedev, 2020). This reference formed the basis for variant calling. A set of the largest 45,000 clusters was then selected based on the completeness and duplication rate of the BUSCO score (74% completeness with 10% duplication rate). This final reference, consisting of 45,000 sequences, was concatenated with the chloroplast genome of *A. ampeloprasum* (NC_044666.1), serving as a reference for subsequent read mapping and filtering (Filyushin et al., 2019). IsoSeq reads of samples 2018‐94‐02 and MS17169‐29‐2 were mapped to the reference transcriptome using Minimap2 v2.11‐r797 (Li, 2018) with additional settings (splice, ‐secondary = no, ‐C5 ‐06, 24, ‐B4). Illumina reads of samples MS 17169‐29‐2, MS 34012‐7, and 2018‐94‐1‐1 were mapped to the reference transcriptome using STARv020201 (Dobin et al., 2013) with default settings. Duplicate read pairs were marked with Picard tools (v2.2.1) MarkDuplicates (https://broadinstitute.github.io/picard/). The bam files were screened with samtools (Danecek et al., 2021) to filter for non‐primary and supplementary alignments (sam flag ‐F2304). NGSEP3 v4.01 (Tello et al., 2019). MultisampleVariantsDetector was used for variant calling of the parents of the two segregating populations (default settings with ‐ploidy = 4). Biallelic single‐nucleotide polymorphism (SNP) variants were selected having a flanking sequence of at least 30 bases without additional SNPs or indels. Probes with a low complexity sequence, low GC content, having an A/T or C/G variant, or showing redundancy were subsequently discarded. For the design of the SNP array, three priorities were defined: Priority 1 focused on probes that needed to be called in both sets, Priority 2 prioritized probes called in the parents of the first cross, and Priority 3 prioritized probes in the parents of the second cross. Following the probe design, a draft de novo assembly of a non‐purged genome became available that was used to filter the recommended probes. For this, the mapped probes were screened with Bowtie2 (–very sensitive) (Langmead & Salzberg, 2012) over possible intron/exon boundaries. Probes with more than the expected maximum of four possible hits in a tetraploid individual were filtered out. A maximum number of 350 probes per contig were chosen.

### 2.7 Dosage calling

The array hybridization was performed in two separate experiments, one with each of the two F1 populations, along with replicate samples of the parents, and in the case of the second F1 population, also with unrelated material of different leek types. Dosage calling was performed with the R package fitPoly (Voorrips et al., 2011; Zych et al., 2019).

### 2.8 Linkage mapping

Linkage mapping in both F1 populations was performed using the R package polymapR (Bourke et al., 2018), according to the vignette of that package. The procedure consists of the following consecutive steps: filtering against markers and SNPs with too many missing data, merging duplicate F1 individuals, binning duplicate markers, assigning simplex × nulliplex (and analogous) markers to chromosomes and then to homologues for the two parents separately, matching the maternal and paternal chromosomes using simplex × simplex markers, assigning all other marker types to the simplex–nulliplex homologues, ordering the markers on the chromosomes, and fine‐tuning by successively eliminating ill‐fitting markers. Finally, the duplicated markers that were set aside in the binning step were added back at the position of the marker representing the bin.

A consensus linkage map over the two populations was created by combining the pairwise linkage data (i.e., for each marker pair the recombination fraction and the log of odds [LOD] score) of the corresponding chromosomes from both populations. For marker pairs that occurred in the linkage data of both populations, the combined recombination and LOD score was calculated by averaging the separate values, weighted by the squares of the LOD scores. With the combined linkage data, marker ordering and fine‐tuning were performed again.

Diagnostic plots were produced, and a test for preferential pairing was performed, using functions from the polymapR package. Linkage maps were plotted using MapChart (Voorrips, 2002).

### 2.9 Scaffolding

Selected contigs from the purging step were used as a reference for mapping of the probe sequences of Axiom markers present on the final map. The probes were mapped using GMAP v2021‐08‐25 (Wu & Watanabe, 2005) with a large index, suitable for a genome larger than 232 Mb. They were then filtered to ensure a coverage of at least 0.98, an identity of at least 0.95, and only one mapping position. The mapping was combined with the original map and subsequently scaffolded using ALLMAPS v4 (Tang et al., 2015). Public reference genomes of *A. sativum*, *A. fistulosum*, and *A. cepa* were obtained from NCBI RefSeq entries GCA_014155895.2, GCA_030737875.1, GCA_030737815.1, and GCA_030765085.1. Mapping of *A. porrum* markers against those assemblies was done similarly to the mapping described for scaffolding.

### 2.10 Annotation

Repeats in the genome assembly were annotated using the RepeatModeler, RepeatClassifier, and RepeatMasker tools using the combined RepBase (2014) and Dfam (2020) databases for classification of identified repeats (Bao et al., 2015; Hubley et al., 2016; Smith et al., 2013). Full‐length non‐concatemer IsoSeq reads were mapped against the genome assembly using minimap2 *‐ax splice ‐uf –secondary =* *no ‐C5*. The Braker v3 pipeline was then used for building gene models, gene prediction, and gene annotation (Gabriel et al., 2021; Kovaka et al., 2019; Pertea & Pertea, 2020; Quinlan, 2014; Stanke et al., 2008, 2006). In addition, ab initio gene models were predicted with Helixer (Stiehler et al., 2021). Evidence‐driven (Braker 3) and ab initio gene prediction (Helixer) outputs were subsequently used for gene distribution analyses. Efforts to reveal the centromeres, (sub‐)telomeres, and ribosomal DNA arrays were based on BLASTn searches using NCBI accessions MT374061.1, MT374062.1, MH017541.1, and MH017541.1 (Fu et al., 2019; Kirov et al., 2020). Ribosomal DNA arrays were annotated using Infernal v1.1.5 (Nawrocki & Eddy, 2013) and selected eukaryote 5S, 5.8S, 18S, and 28S sequences from the RFAM database.

## 3 RESULTS

### 3.1 De novo assembly

Sequencing of Leidse prei 2018‐94 DNA yielded 883 Gbp of PacBio HiFi data divided over 57.5 million reads (Table S1, Figures S3 and S4). In total, 4.2% and 5.0% of the hits were to plastid and mitochondrial databases, respectively. Quality control showed an average read quality of Q30, which is generally considered a sufficient accuracy level for faithful genome reconstruction. *k*‐mer analysis for *k*‐mer sizes of 21 and 48 revealed a GenomeScope profile showing the highest peak at a coverage of 14 and two additional minor peaks at 28 and 42 (Figure S5), representing genomic sequences present in a single copy or two and three copies, respectively. Modeling of the genome parameters such as genome size, heterozygosity, and repetitiveness using the obtained distribution proved to be unsuccessful as the peaks did not diverge sufficiently, and only one broad peak was captured by the Genomescope2 model. For *k* = 48, GenomeScope2 was also not able to model and properly capture the *k*‐mer histogram (Figure S4B). Nevertheless, *k*‐mer analysis revealed strong AAAB and AB signals, confirming the autotetraploid history of leek (Figure S6). A rough estimate of the genome size based on the total dataset size and the first peak, under the assumption of full heterozygosity, was a 4*n* complement of 63 Gbp with 1*n* equaling 15.75 Gbp.

De novo assembly resulted in a total assembly size of 70.7 Gbp, consisting of 426,980 contigs with an N50 of 3.7 Mbp (Table 1). Primary and alternative assemblies sized to 38.9 and 31.9 Gbp with N50 sizes of 27.5 and 0.14 Mbp, respectively. The largest contigs with an L50 index of 377 in the primary assembly further supported the assignment of the primary assembly (Figure S7). Contamination screening identified 49 and 2 hits to proteobacteria for primary and alternative assemblies, respectively, which were removed. Additionally, 543 and 231 PacBio adaptor and primer sequences were detected in the primary and alternative contig sets, and contigs were consequently either trimmed or broken. Single‐copy ortholog benchmarking, referencing the embryophyta lineage set, showed a very high completeness score of 97.2% but also a high duplication rate of 95.2% as can be expected from a polyploid. For successful scaffolding with a linkage map, marker sequences must map to a single position in the genome, and polyploid‐derived duplication must be minimized. To satisfy this criterion, we applied custom purging based on the BUSCO gene set, selecting 369 contigs (totaling 16.1 Gbp) while maintaining a high benchmark completeness score and reducing the duplication rate to 38.3%. While still suboptimal, this purging result combined with further selection on uniquely mapping markers enabled us to scaffold a pseudohaploid genome. The mapping rates of IsoSeq full‐length transcripts against both the primary and purged contig sets were 96%, indicating that purging did not significantly impact transcriptome representation. This ensured that scaffolding and the final assembly retained high genomic integrity and transcript completeness.

**TABLE 1:** Assembly statistics across different stages of nuclear genome assembly and of chloroplast assembly. Metrics reported include assembly size, contiguity (contig count, N50/L50, maximum contig length), GC content, gap number, BUSCO completeness, and IsoSeq mapping rate.

Assembly	Full	Primary	Alternative	BUSCO‐purged	Scaffolded‐all	Scaffolded‐chromosomes	Chloroplast
Total size	70,789,634,589	38,888,933,560	31,900,701,029	16,186,646,902	16,186,677,702	15,261,338,595	152,493
#Contigs	426,980	59,335	367,645	369	61	8	1
N50	3,760,608	27,546,430	142,303	56,398,703	1,910,501,443	1,910,501,443	152,493
L50	2020	376	35,492	96	3	3	1
GC‐%	35.64	35.61	35.67	35.56	35.56	35.56	36.73
#N	0	0	0	0	30,800	30,800	0
Max contig size	219,647,503	219,647,503	21,158,572	219,647,503	2,446,554,542	2,446,554,542	152,493
BUSCO‐total	1614	1614	1614	1614	1614	1614	NA
Complete	1568 (97.2%)	1560 (96.7%)	1393 (86.3%)	1560 (96.7%)	1560 (96.7%)	1521 (94.2%)	NA
Single	32 (2.0%)	90 (5.6%)	393 (24.3%)	942 (58.4%)	942 (58.4%)	986 (61.1%)	NA
Duplicated	1536 (95.2%)	1470 (91.1%)	1000 (62.0%)	618 (38.3%)	618 (38.3%)	535 (33.1%)	NA
Fragmented	7 (0.4%)	12 (0.7%)	4 (0.2%)	12 (0.7%)	12 (0.7%)	9 (0.6%)	NA
Missing	39 (2.4%)	42 (2.6%)	217 (13.4%)	42 (2.6%)	42 (2.6%)	84 (5.2%)	NA
IsoSeq map rate	NA	96%	NA	96%	96%	96%	NA

Abbreviation: NA, not applicable.

Assembly of the organellar genomes was successful for the chloroplast but not for the mitochondrion. The reference‐based chloroplast assembly has a total length of 152,493 bp and follows the conserved pattern of a long single‐copy section and short single‐copy section interspersed by an inverted repeat. This result indicates that the chloroplast genome is highly conserved and structurally resembles known chloroplast reference genomes. This allowed for successful assembly through reference‐based methods, whereas the failure to assemble the mitochondrial genome may suggest greater complexity or divergence in its structure, possibly requiring more specialized assembly approaches or higher‐quality data.

### 3.2 Linkage mapping

The linkage maps of both F1 populations, and the integrated map, consisted of eight linkage groups, corresponding to the eight chromosomes of leek. Overall statistics of the maps are shown in Table 2. Diagnostic plots for the integrated map are shown in Figure S8, and scatterplots of the Population 1 and Population 2 maps versus the integrated map are shown in Figure S9. The integrated map and the F1 Population 1 map, the latter having the target Leidse prei 2018‐94 plant as father, were quite similar, although the integrated map contains more markers (Table S3 and S4). The length of the chromosomes in both maps did not differ much. The map of F1 Population 2 showed some notable differences with the integrated map and the Population 1 map. The map of Population 2 was much sparser, and chromosomes 6 and 8 in this map were much shorter than in the other maps. Also, the lengths of chromosomes 3 and 7 were larger than those of the other two maps, containing larger gaps. These map features point to a lower quality for the map constructed from the second population. The integrated map of chromosome 6 is based only on the linkage data of F1 population 1; it is identical to it, except that a few markers that were added in Population 2 showed an identical segregation as the markers present in the Population 1 map.

**TABLE 2:** Linkage map statistics for the integrated map and two populations. The number of markers and map lengths (cM) are reported for each of the eight chromosomes, illustrating variation in marker density and recombination distances between populations.

	Integrated map		Population 1		Population 2	
Chromosome	Markers	Length (cM)	Markers	Length (cM)	Markers	Length (cM)
1	1927	137.1	1463	136.8	773	135.8
2	2273	146.3	1593	142.2	1133	147.6
3	1825	138.5	1373	128.4	683	152.9
4	1235	150.9	755	146.7	611	150.0
5	1264	138.3	768	136.9	662	138.6
6	1107	138.5	1090	138.5	557	99.0
7	1115	133.2	833	123.5	445	153.0
8	683	139.8	587	143.1	124	63.7

**FIGURE 1:**
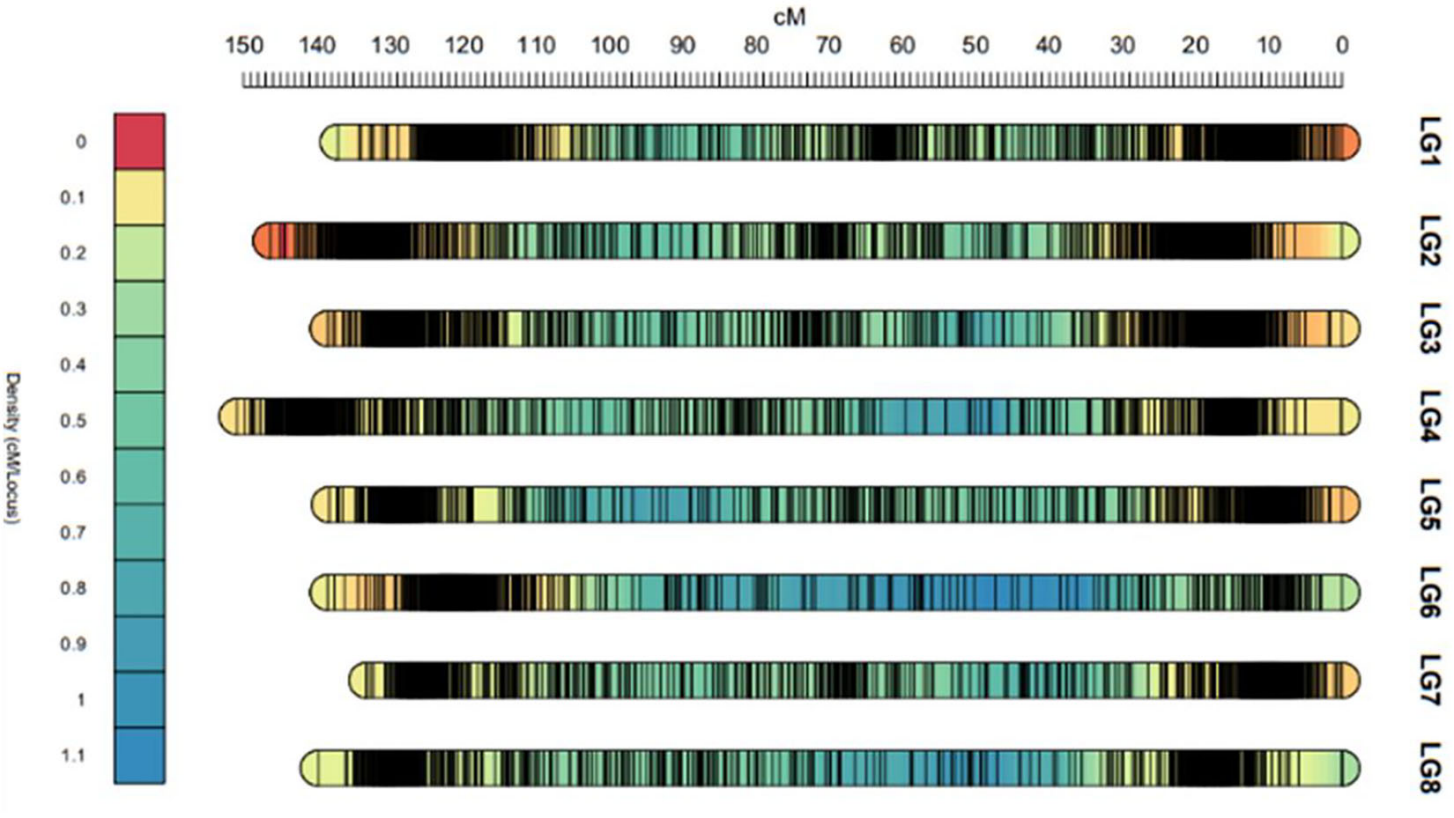
Marker density in centimorgans per locus (cM/locus) across the eight linkage groups of the integrated genetic map of *Allium porrum*. Each vertical line represents a marker. Regions of high marker density appear black and are typically observed at the distal ends and in the centers of the linkage groups.

The markers on the integrated linkage map were mostly concentrated in dense clusters near the ends of all chromosomes (Figure 1). A similar distribution was observed in the separate maps for the first and second F1 populations. However, in the map of the second population, the left distal regions of chromosomes 6 and 8 were missing (not shown). Since the markers were derived from expressed sequences, this suggests that a large part of the expressed genes occurred in blocks with low recombination frequency. In Population 1, the occurrence of double reduction was studied based on simplex × nulliplex markers of both parents. At both telomeres of all chromosomes, a level of 4%–5% double reduction was observed, indicating the occurrence of quadrivalents and polysomic inheritance.

### 3.3 Scaffolding

Mapping of marker sequences to 369 selected contigs, representing a minimal set with maximum BUSCO completeness, showed that 11,143 out of 11,429 markers (97.5%) were successfully mapped. Further filtering based on alignment coverage, identity, and mapping ambiguity removed 835, 300, and 3123 markers, respectively. The remaining 7184 mapped markers were used to orientate and order the 369 contigs with the linkage map as a template to construct a chromosome‐level genome assembly. Of the 316 contigs anchored with the map, 303 could be oriented using at least two aligned markers. The placed contigs represented 94.3% of the total sequence length, with 15.9 Gbp of the 16.2 Gbp scaffolding input mapped to the linkage map. Of this, 15.3 Gbp were anchored to chromosomes, and 15.0 Gbp were also oriented. Unplaced sequences included 25 contigs lacking marker information, and 28 contigs were omitted due to unresolved conflicts with the linkage map. The largest chromosome, chromosome 2, measured 2.45 Gbp, while the smallest, chromosome 8, was 1.40 Gbp. Overall, the correlation between genetic and physical positions was high, with Spearman's rank correlation coefficient (*ρ*) ranging from 0.96 for chromosome 4 to 0.66 for chromosome 6, with most chromosomes exceeding 0.9 (Figure S10 and Table S5).

### 3.4 Repeat and gene annotation

De novo repeat modeling and annotation, supported by RepBase and curated Dfam databases, identified 4312 repeat models. The largest category remained unclassified, including 1725 unknown repeat families and 1688 unclassified LTR retrotransposon families. Among the classified elements, 391 were assigned to the LTR/Copia family and 361 to the LTR/Ty3 family (formerly known as Gypsy). In total, repeat masking accounted for 81.51% of the leek genome assembly, underscoring its highly repetitive nature, dominated by both known and novel transposable elements.

Gene annotation of the repeat‐masked genome predicted 66,021 genes, with an average gene length of 6553 bp and a mean of 3.7 exons per gene (Table S6). Analysis of repeat and gene distributions, based on both evidence‐driven and ab initio predictions, revealed a generally uniform distribution along the physical chromosomes, with no clear enrichment at centromeric or telomeric regions (Figure S11).

### 3.5 Scaffolded genome reveals distinct recombination landscape

**FIGURE 2:**
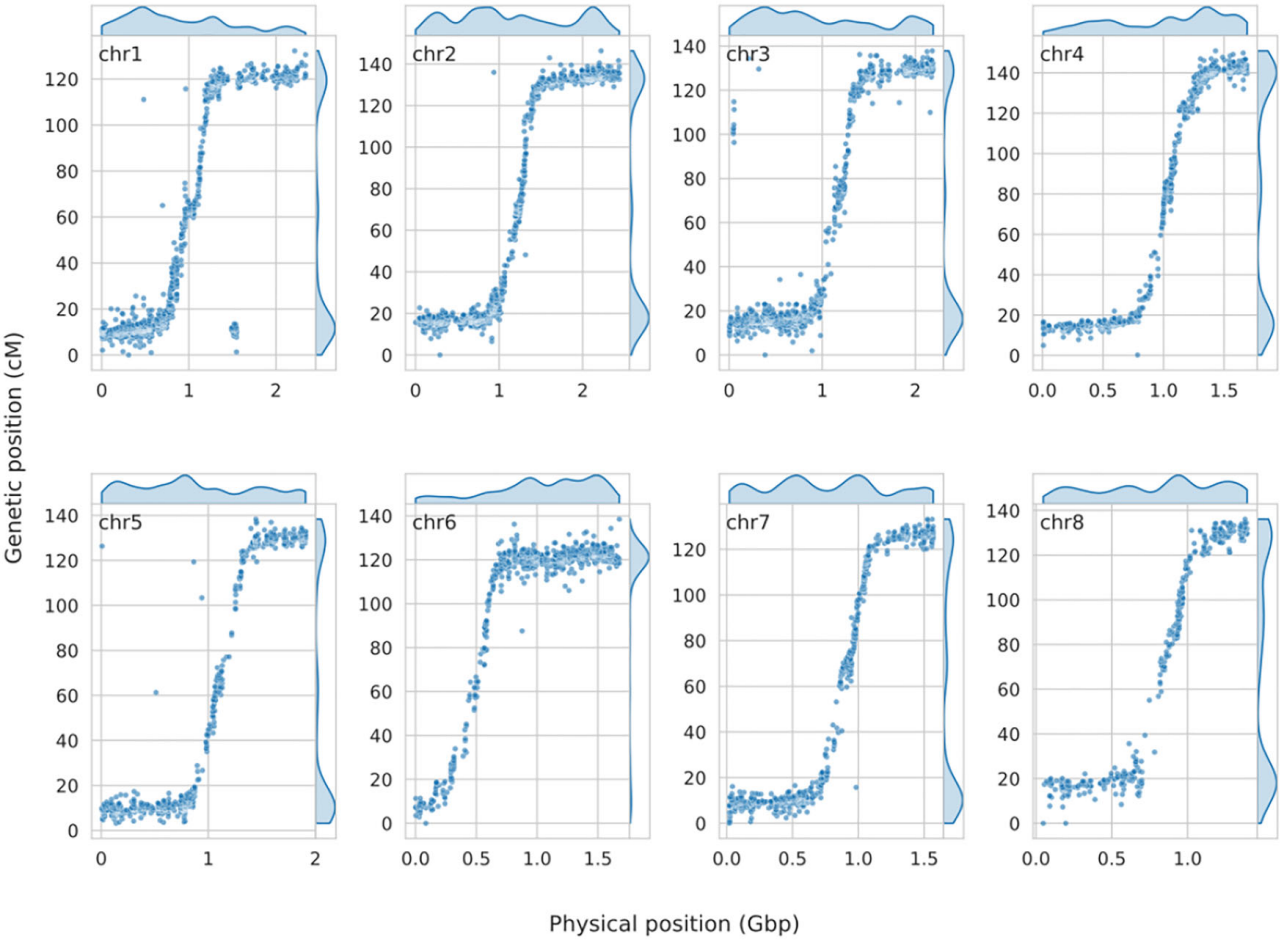
Marey maps showing the relationship between physical (*x*‐axis) and genetic (*y*‐axis) positions of markers. Blue dots indicate genetic marker positions. Density plots along both axes summarize the distribution of markers for each chromosome. The sigmoid‐like patterns reflect the recombination landscape in *A. porrum*. Density along the *y*‐axis highlights clusters of markers in regions with low recombination, whereas physical positions are more uniformly distributed across chromosomes. Notably, genetic positions are shifted toward the proximal ends of the chromosomes, emphasizing the bias introduced by reduced recombination in these regions.

The Marey maps (Figure 2) show a comparison between the generated linkage map and the physical map that was obtained by de novo assembly. Overall, the maps indicated a sufficient coverage of markers along the chromosomes. The densely populated areas on the linkage map made up the chromosome ends. Because the array design was based on transcriptome‐derived variants, the marker distribution showed the density of genic regions, which are present quite uniformly along the physical chromosomes in leek (with slightly higher densities in distal parts of the linkage map). For all chromosomes, a full sigmoid‐like pattern could be observed, with the exception of chromosome 6, showing a partial sigmoid‐like pattern with a flat part extending to the chromosome end. Most of the chromosomes lacked markers in the middle section, possibly representing the centromere region. The flatter parts at the chromosome ends indicated a smaller distance between successive markers on the genetic map, while the middle parts of each chromosome showed relatively larger genetic distances between successive markers. This pattern thus pointed to a relatively high recombination frequency in the middle of the chromosome compared to the chromosome ends. Apparently, recombination in *A. porrum* occurs mainly in the regions directly adjacent to the centromere and not in the distal chromosome ends, consistent with the cytological observation that chiasmata in *A. ampeloprasum* are predominantly localized proximally to the centromere (Kollmann, 1972).

### 3.6 Marker mapping to *A. fistulosum*, *A. sativum*, and *A. cepa*

**FIGURE 3:**
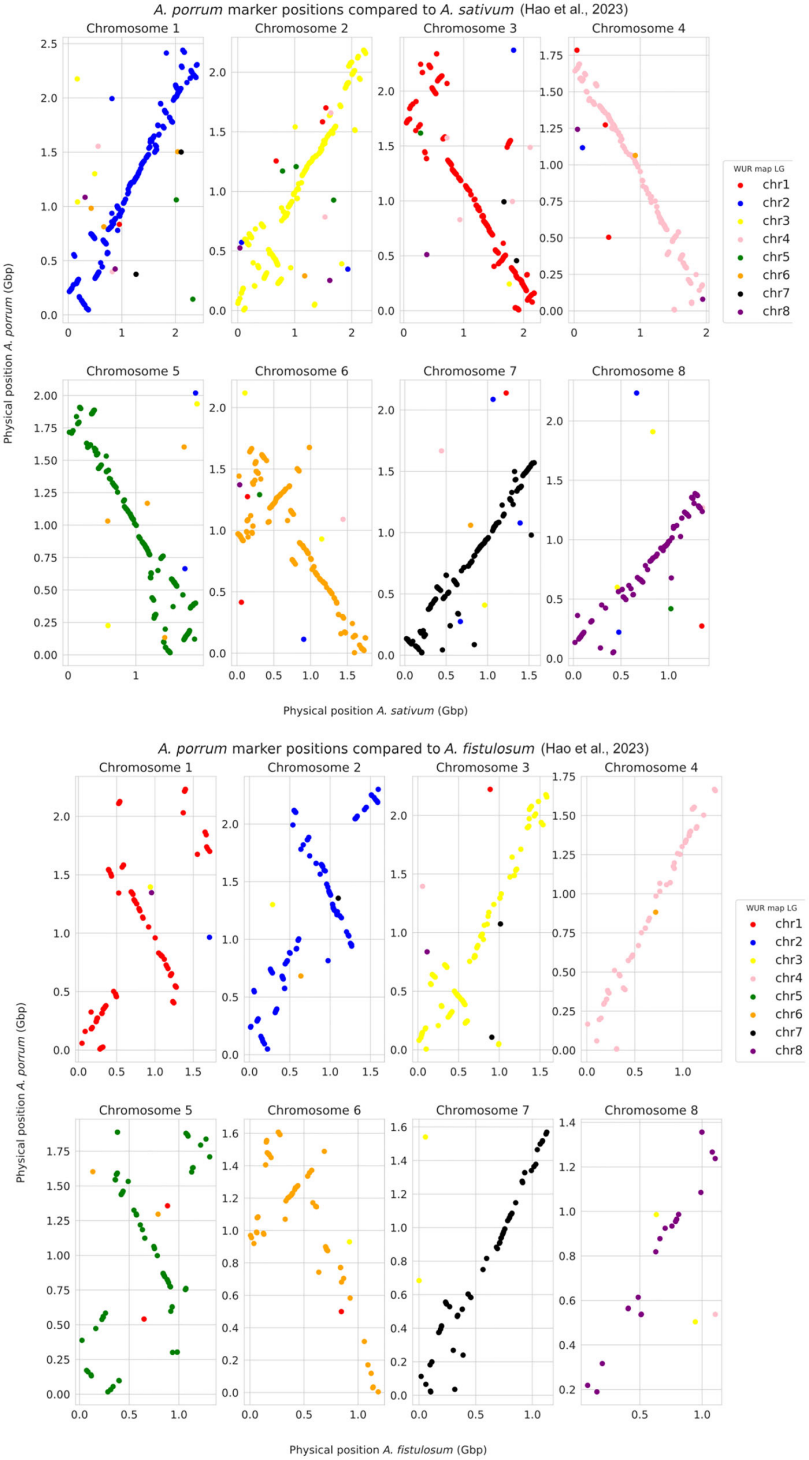
Mapping of markers on *A. porrum* (*y*‐axis) and *A. sativum* (upper panel) (*x*‐axis) reveals long syntenic stretches but also inversions in the range of 10–100 Mbp. A similar mapping to *A. fistulosum* (lower panel) reveals several larger structural variations such as inversions of ∼1 Gbp on chromosomes 1, 2, and 5. Chromosomes 4 and 7 appear more colinear.

A comparison between the final genetic map of *A. porrum* and mapping positions of our markers on the genome of *A. sativum* (Sun et al., 2020) identified 4785 mappable marker positions, revealing synteny between leek chromosomes 1, 2, 3, 6, and 8 with *A. sativum* chromosomes 6, 3, 8, 4, and 1, respectively. However, these chromosomes also exhibited synteny breaks due to intrachromosomal inversions (Figure S12). Additionally, large interchromosomal rearrangements were observed for leek chromosomes 4, 5, and 6. Specifically, leek chromosome 2 was partially syntenic with *A. sativum* chromosomes 2 and 7, leek chromosome 5 showed partial synteny with *A. sativum* chromosomes 2 and 5, and leek chromosome 6 was partially syntenic with *A. sativum* chromosomes 5 and 7. However, a comparison with *A. sativum* from Hao et al. (2023) identified 5013 shared markers but did not reveal intrachromosomal rearrangements (Figure 3). This discrepancy may reflect differences in genome assembly quality, as suggested by Hao et al. (2023). Alternatively, it is possible that these differences result from accession‐specific rearrangements in *A. sativum*. A comparison with *A. cepa* (Hao et al., 2023) identified 1116 shared mappable markers and revealed synteny breaks due to intrachromosomal rearrangements across all chromosomes (Figure S12). In contrast, no major interchromosomal rearrangements were observed between leek and *A. cepa*.

We also compared the physical marker positions of our map with those of the map of *A. fistulosum* (Welsh onion/bunching onion) (Hao et al., 2023; Liao et al., 2022). This yielded 1135 unambiguous mapping positions. As shown in Figure S12, large intrachromosomal inversions are present on chromosomes 1, 2, 3, 5, and 6. Although a few markers suggest possible translocations by mapping to different chromosomes—indicated by deviations in the color code displayed in the chromosome synteny panels of Figures 3 and Figure S12—no major interchromosomal rearrangements were observed between leek and bunching onion. Considering the higher number of shared markers and the smaller size of inversions, *A. porrum* may be more closely related to *A. sativum* than to *A. cepa* or *A. fistulosum*. While these findings suggest a possible closer relationship, they should be interpreted with caution, as marker data and chromosomal rearrangements alone provide a limited view of phylogenetic relatedness. Nevertheless, our observations are in line with the relationships among *Allium* species reported by Hirschegger et al. (2010), though additional molecular and genomic evidence would be required to substantiate this hypothesis.

## 4 DISCUSSION

### 4.1 First chromosome‐scale reference genome of *A. porrum*

The first genome sequence of a highly heterozygous tetraploid *A. porrum* is a valuable addition to the collection of Allium genomes that have currently been reconstructed, as the current collection does not contain such a complex genome yet. The total size of the 15.26 Gbp anchored genome is slightly smaller than the recently published genomes of onion (15.78 Gbp) and garlic (15.52 Gbp), but larger than the Welsh onion genome of 10.48 Gbp. Compared to the N50 contig sizes of 81.66, 109.82, and 507.27 Mbp for onion, garlic, and Welsh onion, respectively, the N50 leek contig size of 57.37 Mbp is notably smaller. This is partly due to the relatively low coverage per haplotype, combined with the high heterozygosity and possibly a smaller sequence library insert size. Since the Allium genomes reported by Hao et al. (2023) were also constructed by HiFi‐based technology, we regard the difference in N50 contig size as less likely to be the result of the sequencing technology used. Several findings have reported on the genome size for *A. porrum*. Our leek genome assembly size significantly exceeds the flow cytometric values reported by Arumuganathan and Earle (1991), who found a 2C value of 50.27 pg for *A. ampeloprasum*, corresponding to a single‐copy genome size of 12.29 Gbp. Ricroch et al. (2005) reported that the genome size of tetraploid *A. porrum* is 50.7 ± 0.7 pg, corresponding to *n *= 12.45 Gbp, and additionally noted that the diploid *A. ampeloprasum* has a haploid genome size of 16.37 Gbp. Ohri et al. (1998) summarized findings on tetraploid *A. porrum*, which range from 11.78 to 31.93 Gbp for a single genome copy. Overall, the size of the leek genome assembly we report here falls within the previously reported genome size ranges for both *A. ampeloprasum* and *A. porrum*.

The use of PacBio HiFi reads with an average read quality score of Q30 was a key factor contributing to the high quality of the reconstructed leek genome. The high quality of the presented assembly is substantiated by the high completeness of conserved single‐copy ortholog (BUSCO) genes. The 97.2% completeness slightly exceeds the benchmark results for onion (96.4%), garlic (92.6%), and Welsh onion (96.6%) genomes. Our annotation efforts show that the number of 66k genes in leek is in the same range as found by *Hao* in garlic and onion, which are similarly sized genomes. Annotation of repetitive sequences is different, however, as the percentage of the genome marked as repetitive is around 11–13 percentage points lower. Marker‐based scaffolding with probe sequences designed from transcriptome data and BUSCO‐based purging are possible causes for the exclusion in the scaffolding process of contigs consisting mainly of repeat sequences. This depletion would lower the total repeat sequence relative to the total assembly size. In addition to the high BUSCO scores, the high collinearity with an integrated map from two mapping populations confirms that the order of markers on the generated contigs aligns precisely with linkage calculations. Furthermore, the successful anchoring and orientation of a significant proportion (94.3%) of the genome assembly onto the integrated linkage map, along with the high correlation between genetic and physical positions, demonstrates the robustness and accuracy of the assembly process. The few unresolved contigs and lower correlation on chromosome 6 suggest areas for further refinement, but overall, the results provide a highly contiguous and well‐ordered chromosome‐level assembly that will support downstream genomic analyses.

### 4.2 Marker‐rich genic regions in distal clusters on linkage maps and recombination predominantly occurring near centromeres

Understanding gene distribution and recombination behavior across a wide range of Allium genomes is crucial for developing effective breeding programs. Furthermore, insight into marker distribution can aid in more efficient breeding by introducing marker‐assisted selection. By leveraging dense markers in gene‐rich areas, breeders can more accurately track desired traits, improving the speed and efficiency of developing new leek varieties. The markers we developed from mapped transcript sequences primarily target expressed leek genes. Surprisingly, we observed a pronounced clustering of markers toward the chromosome ends on the linkage map, whereas recombination predominantly occurred proximal to the leek centromere (Figure 2). This clustering of markers is partly the result of a relatively low recombination frequency toward the ends of the leek chromosomes, as we observed a relatively more even marker distribution on the physical map (Figure 2). We are aware that the use of transcriptome‐derived markers inherently emphasizes genic regions and does not capture the full extent of repetitive DNA, which comprises the majority of the leek genome. However, our Marey map comparisons demonstrate that the resulting marker distribution nevertheless provides a sufficiently representative coverage across all chromosomes to allow reliable inferences about recombination patterns. Importantly, the sigmoid‐like recombination profiles we observed are consistent with cytological evidence of chiasma localization in related species (Kollmann, 1972), which supports the biological relevance of our findings. Thus, while we recognize that the density of markers could be increased by integrating additional repeat‐based or whole‐genome markers, the present dataset provides a robust and biologically meaningful framework for analyzing recombination landscapes in leek. Taken together, this contrast suggests that while breeders may focus on gene‐rich regions for selecting desirable traits, recombination in the distal leek chromosome regions is limited, potentially constraining the reshuffling of traits to new generations. Consequently, understanding the genetic landscape of *Allium* species is essential for optimizing breeding approaches.

Notably, variations in recombination behavior have been observed across different *Allium* species and subspecies. In tetraploid *A. porrum*, Levan (1940) reported a predominantly proximal localization of chiasmata, being cytological manifestations of COs. Further cytological studies by Kollmann (1972) on *A. ampeloprasum* subspecies (*ampeloprasum* and *truncatum*) revealed distinct recombination patterns: while ssp. *ampeloprasum* exhibited chiasmata localized near the centromere, ssp. *truncatum* displayed subterminal or terminal localization. The differences in chiasma localization appeared independent of ploidy level. Profound differences in recombination behavior have also been observed between diploid *Allium* species. In onion (*A. cepa*), COs predominantly occur in the distal chromosome regions, whereas in *A. fistulosum*, they are mainly localized proximally (Emsweller & Jones, 1935b; Khrustaleva et al., 2005; Kudryavtseva et al., 2023). Moreover, chiasmata in an *A. cepa* × *A. fistulosum* hybrid were found to shift significantly toward the distal regions of *A. fistulosum* chromosomes, suggesting genetic control over CO localization. This observation supports the hypothesis of Emsweller and Jones (1935a) that *A. cepa* and *A. fistulosum* possess dominant and recessive genes, respectively, regulating distal and interstitial chiasmata localization. These recombination differences across *Allium* species may have significant implications for breeding and warrant further investigation into Allium meiotic recombination landscapes.

A comparison of our genetic and physical chromosome maps of *A. porrum* (Figure 2) further supports the previously reported findings and indicates that recombination occurs predominantly in the central parts of leek chromosomes, containing functional centromeres as demonstrated previously by Levan (1940). Subsequent FISH analysis in *A. cepa* and *A. fistulosum* showed the co‐localization of repetitive centromeric sequences like those found in many plant species (Kirov et al., 2020), suggesting that recombination in leek also occurs mostly proximally to the centromeres. We were, however, unable to obtain any conclusive results on the inclusion of centromeres in our assembled genome, presumed to be caused by the absence of markers in the centromere itself.

While the pseudohaploid assembly presented here provides a valuable resource, it collapses homologous variation, limiting applications such as allele‐specific expression and haplotype‐resolved analyses. Our study of meiotic recombination provides important insights, though these could be further refined with additional allelic resolution. To address this, we have started construction of a fully phased genome assembly, which will enable resolution of hidden allelic variants and provide a more detailed understanding of recombination and introgression breeding in *Allium*. Finally, in this study, we combined information on recombination frequencies between markers obtained through linkage mapping with their physical genome sequence positions, further confirming the proximal localization of recombination in leek chromosomes and further substantiating the existence of a dichotomy in recombination behavior in *Allium*. To our knowledge, this is the first such comparison in the *Allium* genus.

## AUTHOR CONTRIBUTIONS

**Ronald Nieuwenhuis**: Data curation; formal analysis; investigation; methodology; software; validation; visualization; writing—original draft; writing—review and editing. **Roeland Voorrips**: Data curation; formal analysis; investigation; methodology; software; validation; visualization; writing—original draft; writing—review and editing. **Danny Esselink**: Data curation; formal analysis; investigation; methodology; software; writing—review and editing. **Thamara Hesselink**: Investigation. **Elio Schijlen**: Investigation. **Paul Arens**: Investigation; writing—review and editing. **Jan Cordewener**: Investigation. **Hetty C. van den Broeck**: Investigation. **Olga Scholten**: Conceptualization; funding acquisition; project administration; writing—review and editing. **Sander Peters**: Conceptualization; funding acquisition; project administration; writing—original draft; writing—review and editing.

## CONFLICT OF INTEREST STATEMENT

The authors declare no conflicts of interest.

## Supporting information



Tables S1 HiFi sequencing overview. Contains an overview of per SMRTcell stats on data production
Tables S2 RNA‐seq samples. The samples and tissues used for transcriptome sequencing
Tables S3 Integrated genetic map. The linkage map generated from two populations merged (average) except for chromosome 6
Tables S4 Linkage map stats per LG. Overview of statistics for each linkage group
Tables S5 Spearman rank correlation. Spearman’ s rank correlation (ρ) between genetic and physical order of markers per chromosome
Tables S6 Annotation overview. Overview of annotation statistics per chromosome

Figures S1 Assembly workflow. General overview of assembly process
Figures S2 Custom purging pseudocode. Describes method on how purging of primary contigs was done
Figures S3 HiFi read lengths. Read length distribution of all combined HiFi reads
Figures S4 HiFi mean read quality. Mean read quality distribution of all combined HiFi reads
Figures S5 Genomescope2 output. K‐mer histogram and genomescope2 model fit for k = 21 (A) and k = 48 (B)
Figures S6 Smudgeplot. Smudgeplot for detection of ploidy and ploidization events
Figures S7 L50 plots. Plots showing the contiguity increase for each assembly iteration
Figures S8 Linkage map QC. For each linkage group there are pairwise comparisons of estimated recombination frequency and observed recombination frequencies and LOD scores
Figures S9 Linkage map comparison. Comparison of the separate maps to the integrated map
Figures S10 Scaffolding per linkage group. Shows how each chromosome is built from contigs and the marker order from AllMaps output
Figures S11 Feature density on chromosome level assembly. Visualized the feature density for repeats, genes, markers, ribosomal DNA and some satellite repeats
Figures S12 Structural homology to *A. sativum* and *A. cepa*. Genome comparisons based on marker mappings from our integrated map to two different version of the garlic genome and the onion genome

## Data Availability

The genomic and transcriptomic data used in this study are available under NCBI BioProject accession PRJNA1231124.
