# Supplementary material for: High‐resolution genome and genetic map of tetraploid Allium porrum expose pericentromeric recombination
Source: Plant Genome. 2025 Dec 8;18(4):e70159. doi: 10.1002/tpg2.70159 (PMC12683693; doi:10.1002/tpg2.70159)

# Supplementary Figures

## S1 Assembly Workflow

**Overview of the assembly process for the leek (*A. porrum*) pseudohaploid genome. The process includes the mapping of two populations and applying the constructed linkage map for scaffolding of a long-read (PacBio HiFi) based *de novo* assembly into a pseudohaploid chromosome-scale assembly.**


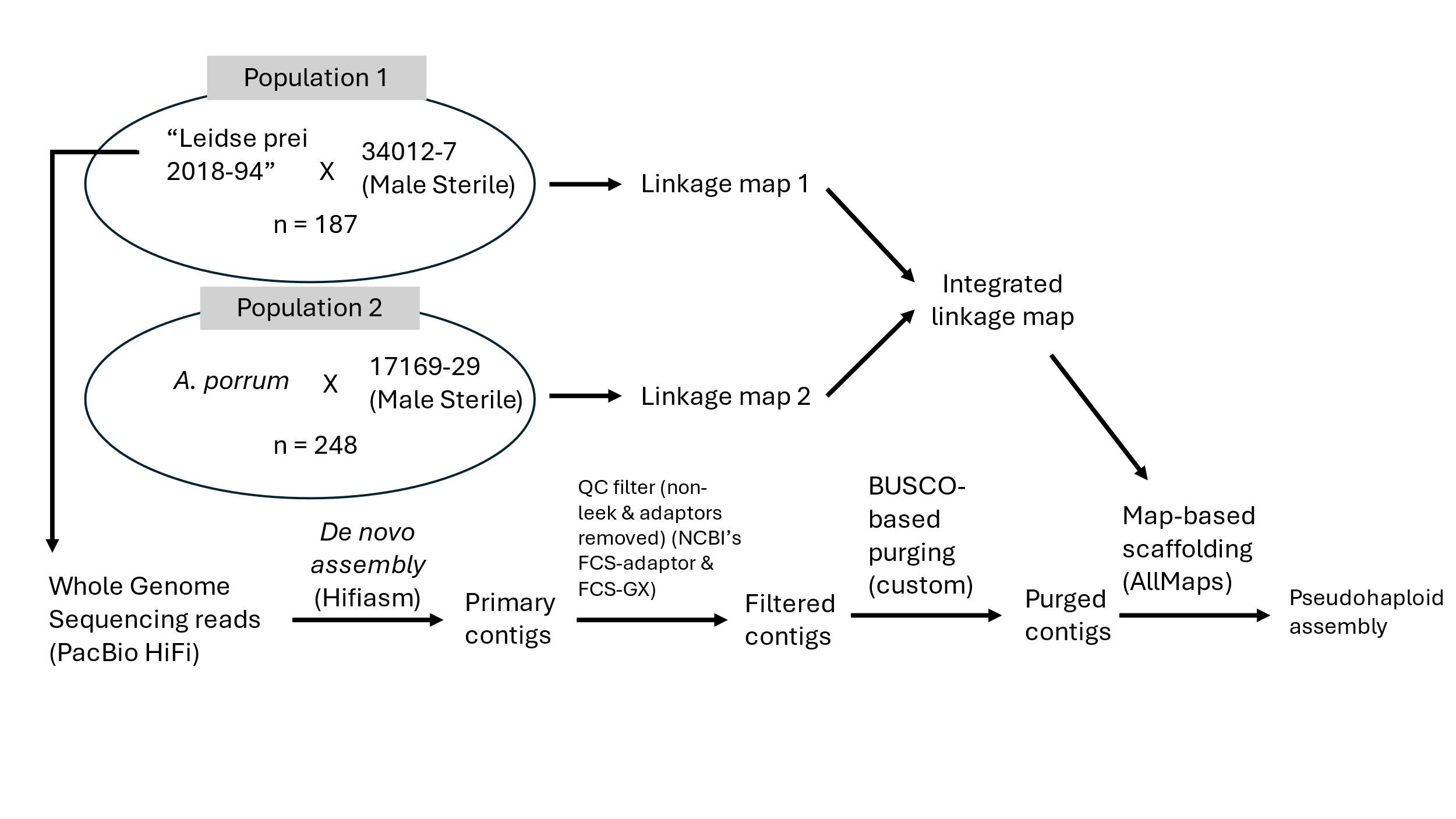


## S2 BUSCO-based purging pseudocode

**Pseudocode illustrating the custom purging process applied to filtered contigs to obtain a minimal set with maximal BUSCO completeness for scaffolding.**

*C=[contigs] #Sorted by contig length in descending order*

*buscos_in_selected = []*

*selected_contigs = []*

*For contig in C:*

*If any busco_on_contig not in buscos_in_selected*

*append busco_gene to buscos_in_selected*

*append contig to selected contigs*

*else:*

*next*

## S3 HiFi read lengths

**Read length distribution of leek PacBio HiFi long reads used for *de novo* assembly.**


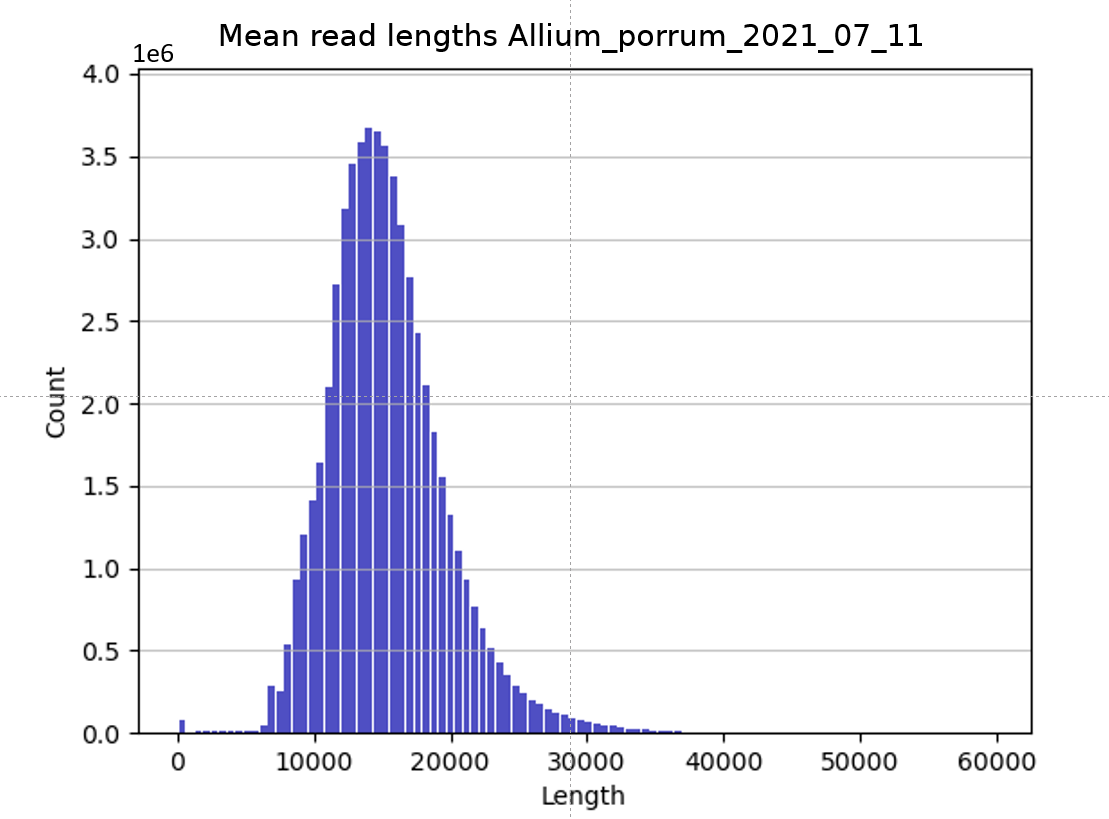


## S4 HiFi mean read quality

**Mean read quality distribution of leek PacBio HiFi long reads used for *de novo* assembly. Quality values (QV) on the x-axis are expressed as PHRED score, which represents a logarithmic transformation of error probability. For example, a QV of 30 corresponds to a 0.1% probability that a base is incorrectly called.**


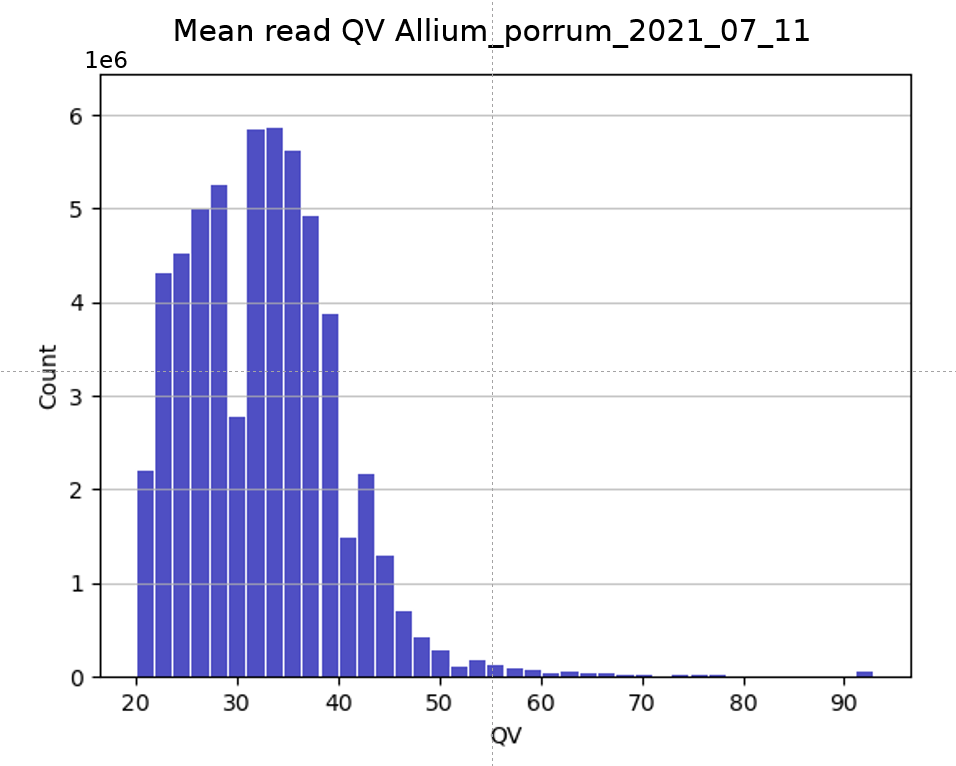


## S5 GenomeScope2 output

**Output of GenomeScope2 analysis for k-mer sizes of 21 (panel A) and 48 (panel B). The blue histogram shows the frequency distribution of k-mers in the leek PacBio HiFi long-read dataset used for *de novo* assembly. The black line represents the model fit overlaid on the histogram, from which genome characteristics are estimated. Because the model did not converge with the observed distribution, the parameter estimates are likely inaccurate, possibly due to insufficient sequencing coverage.**


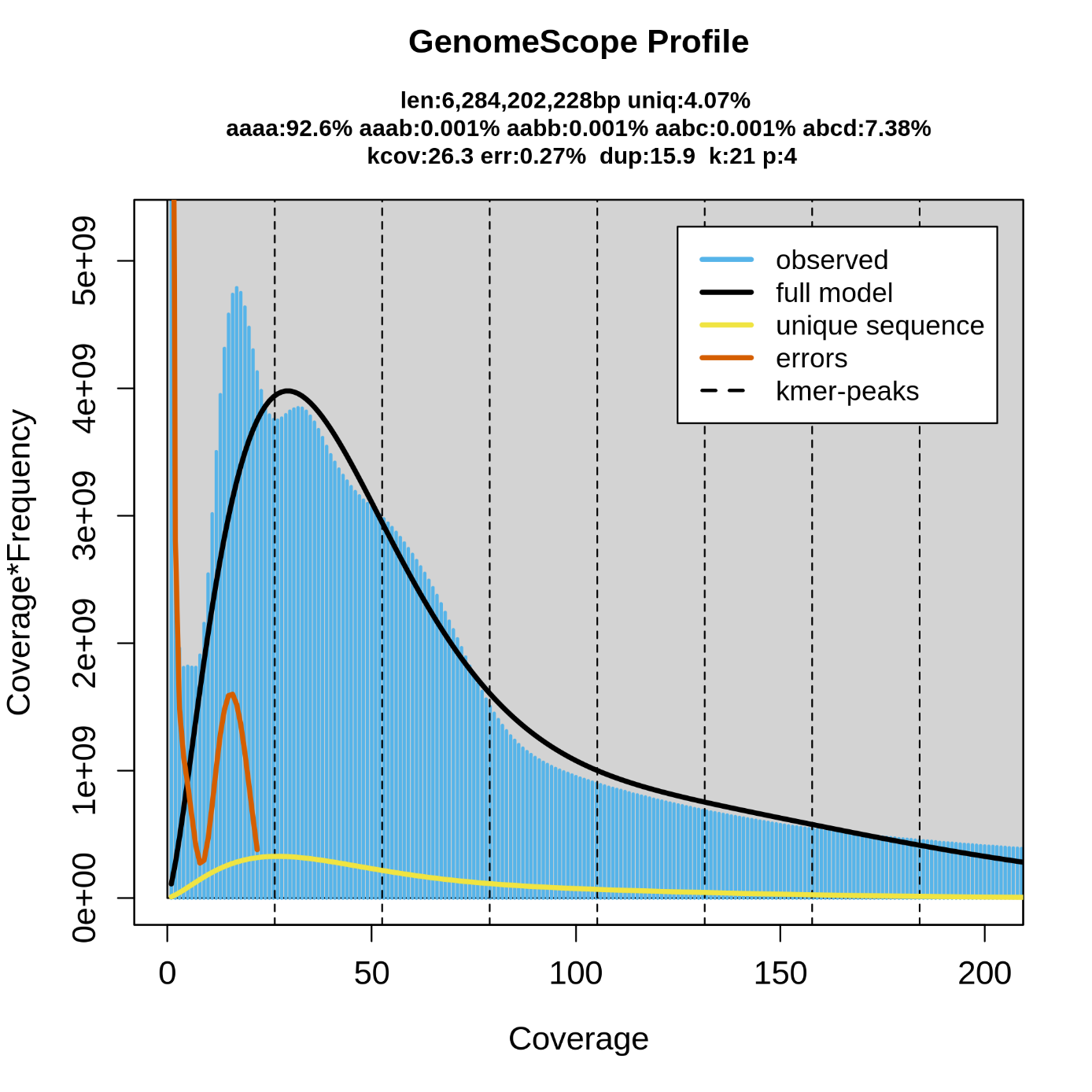


**A**


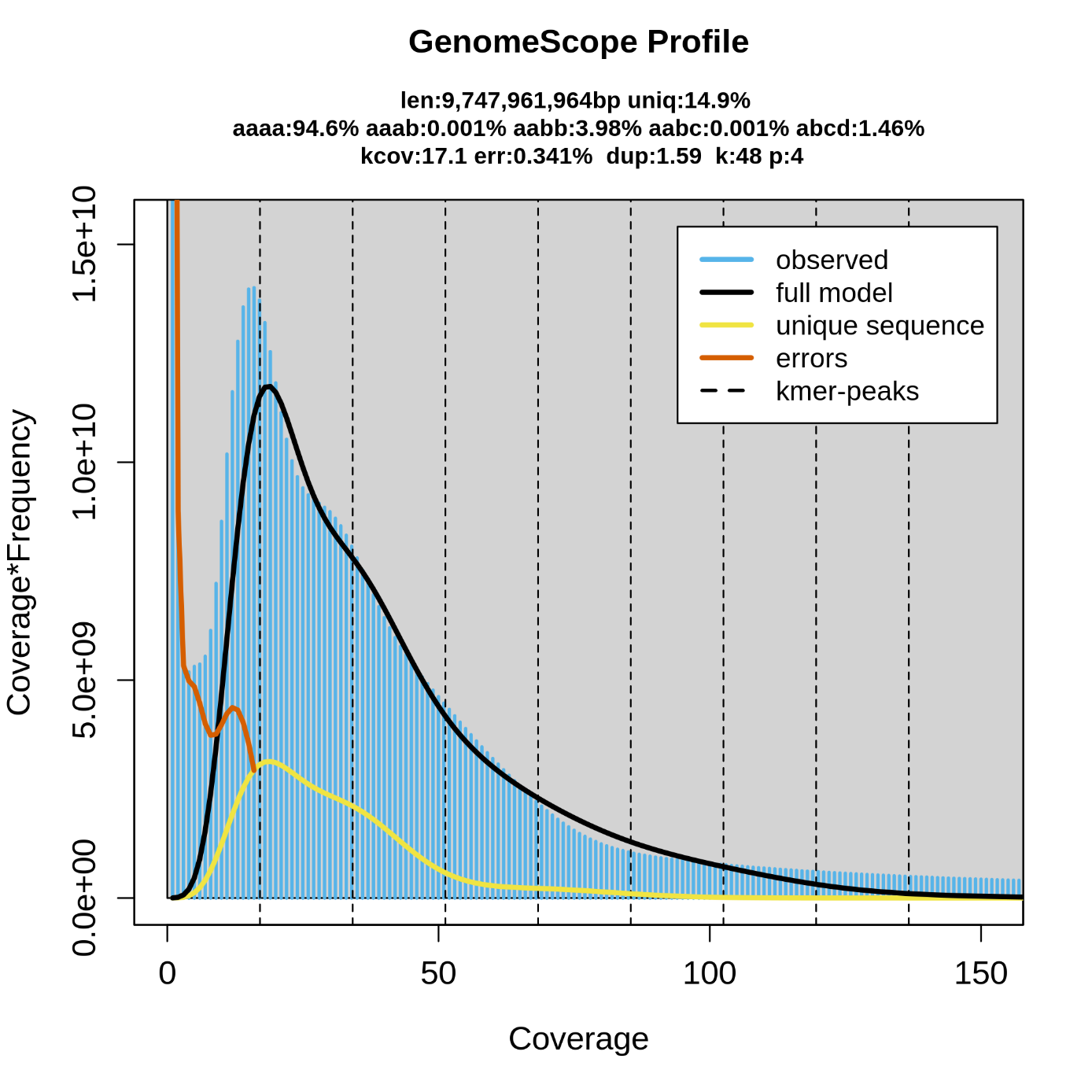


**B**

##
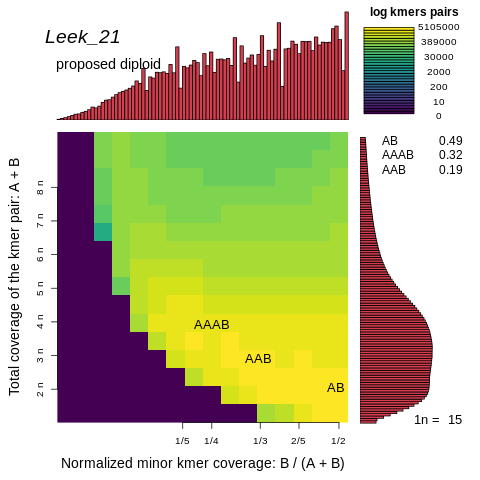

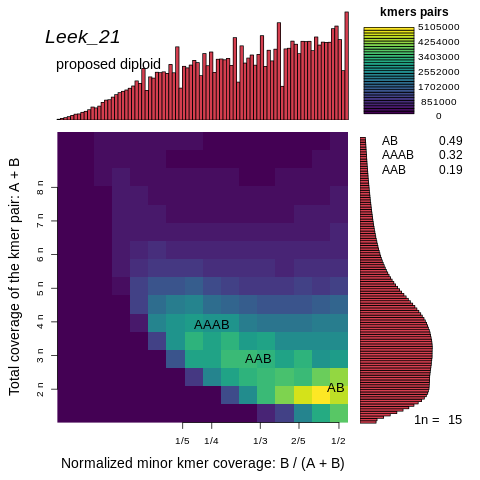
S6 Smudgeplot

**Smudgeplot k-mer pair based ploidy inference.**

## S7 L50 plots

**Cumulative contig size distribution ordered by decreasing contig length. Across successive stages of the assembly process, contiguity improves, with the final stages showing an almost vertical curve, indicating that the assembly consists of fewer but longer sequences.**

##
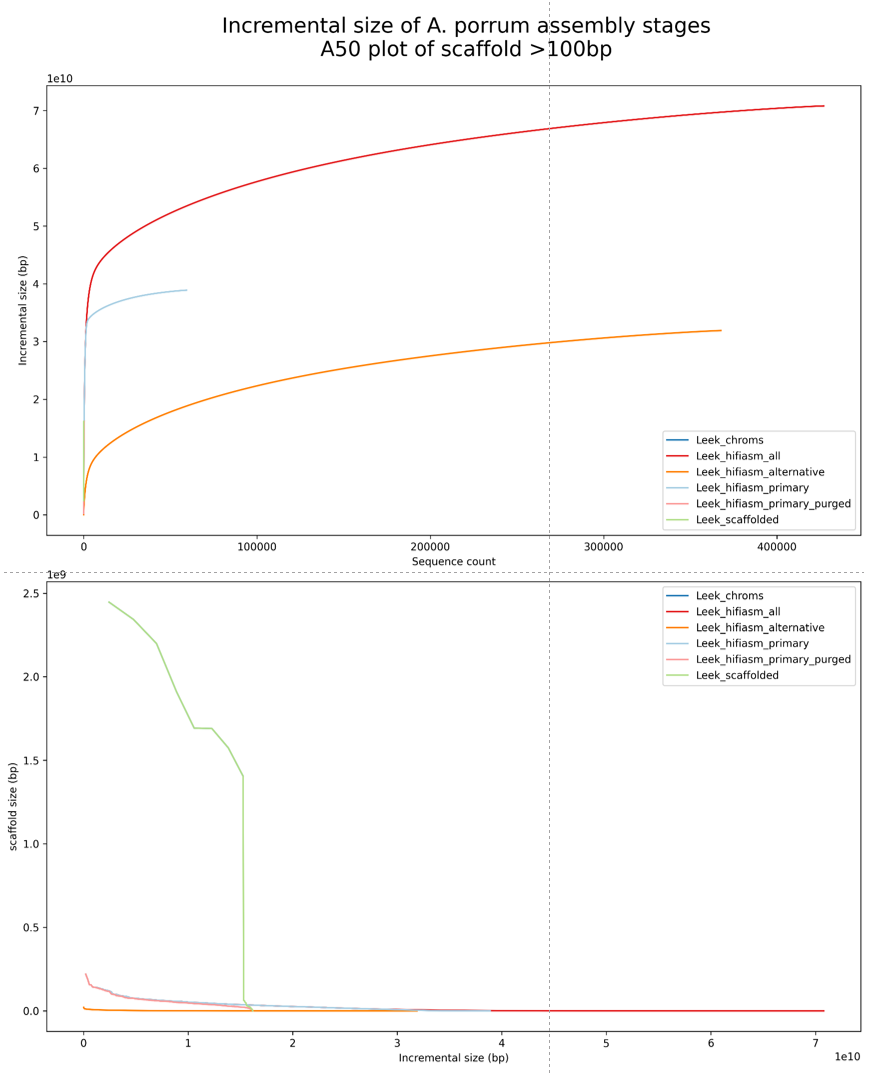
S8 Linkage map QC


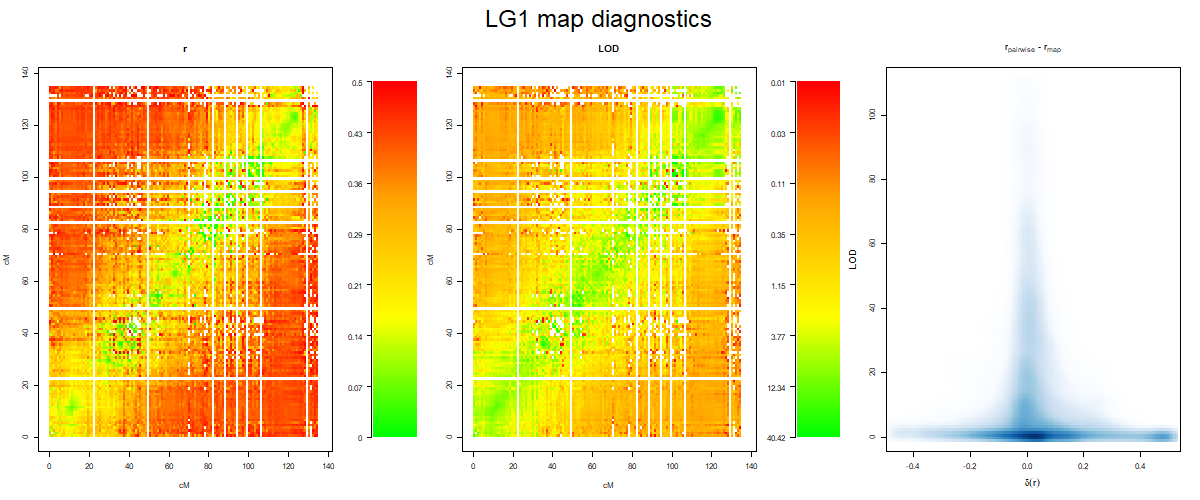

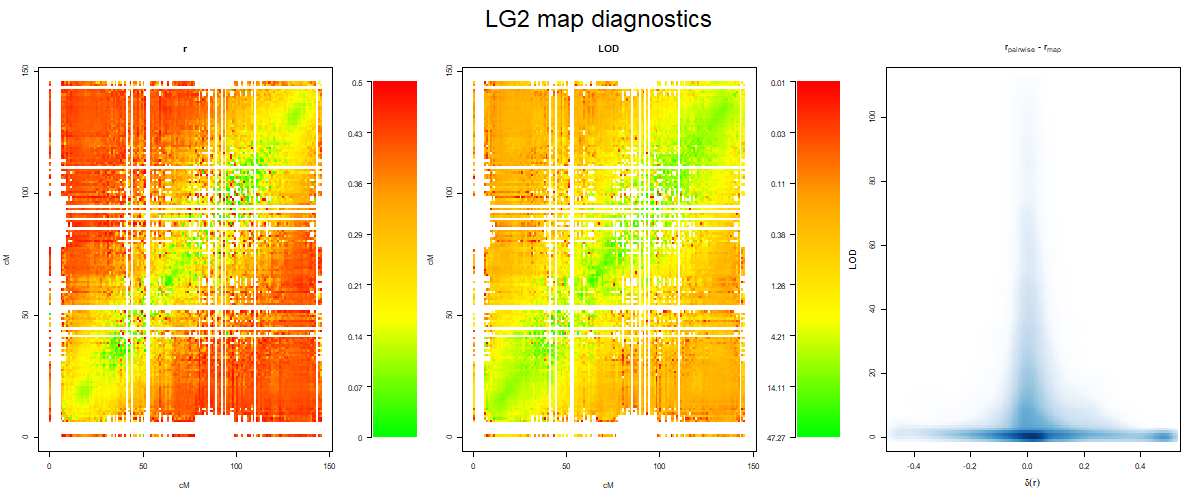

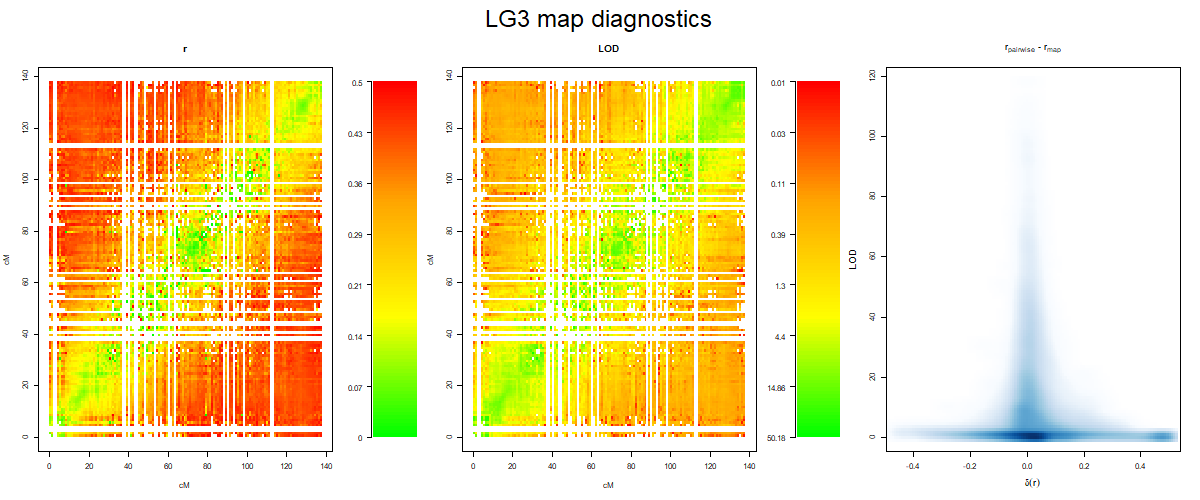

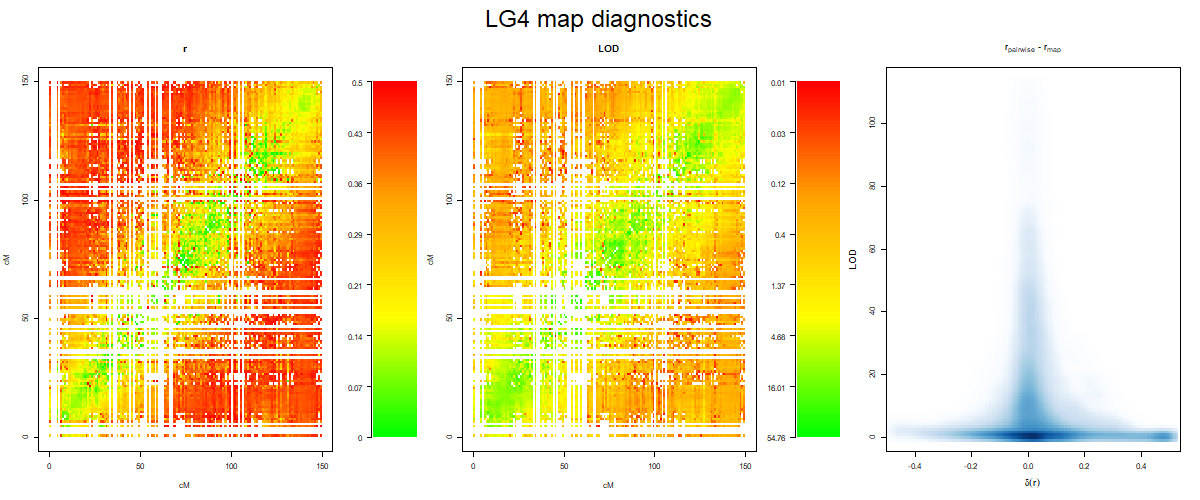

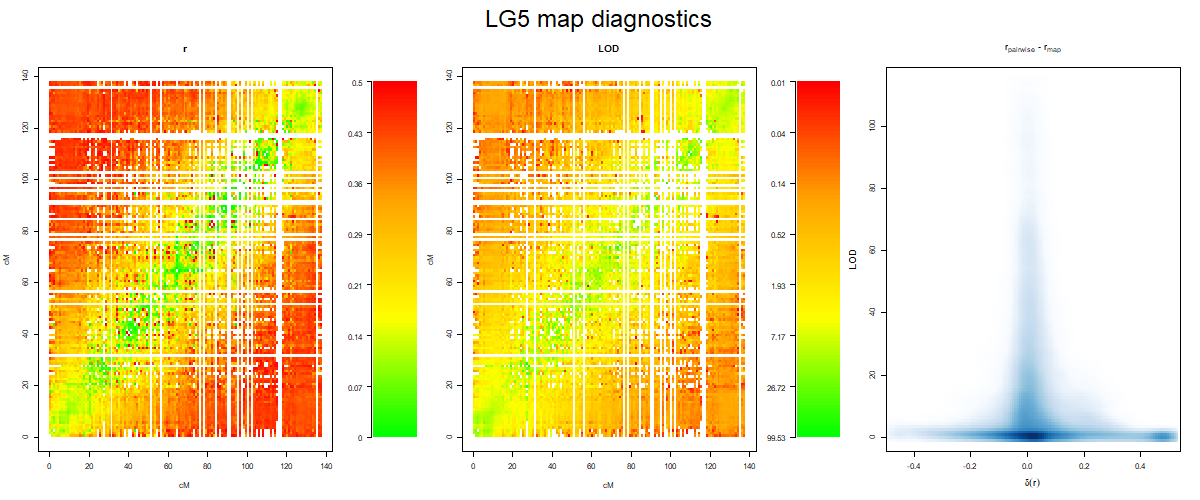

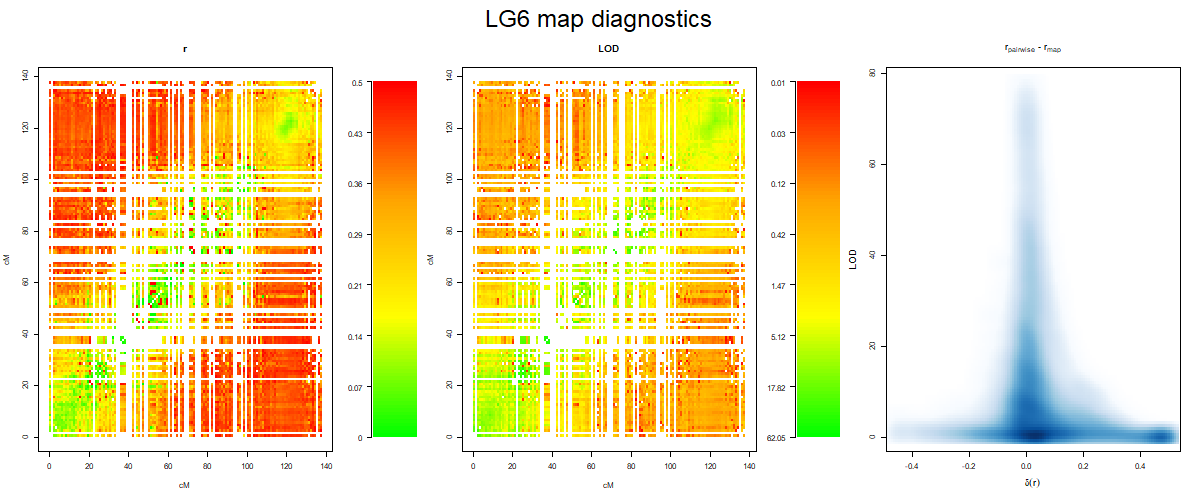

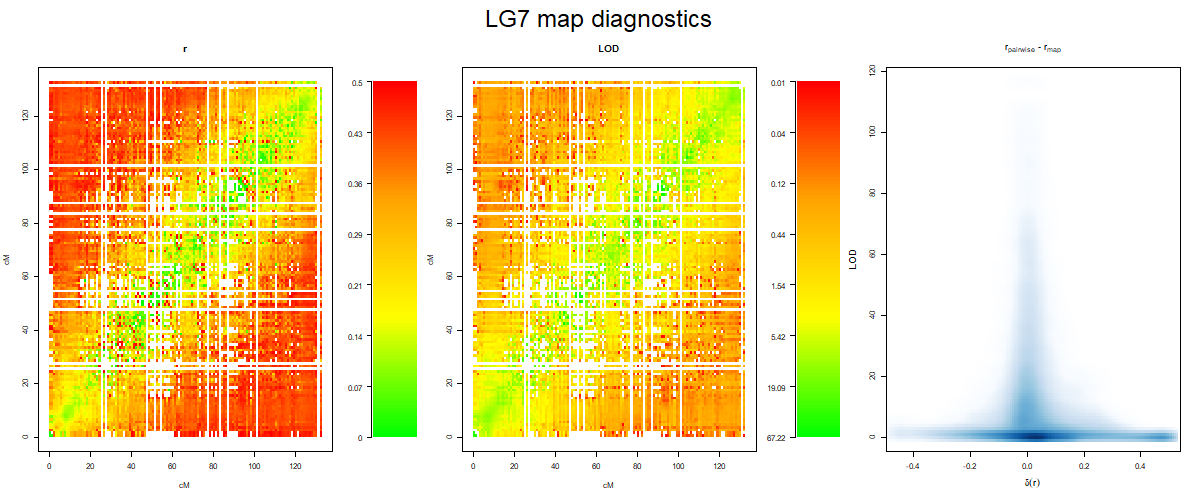

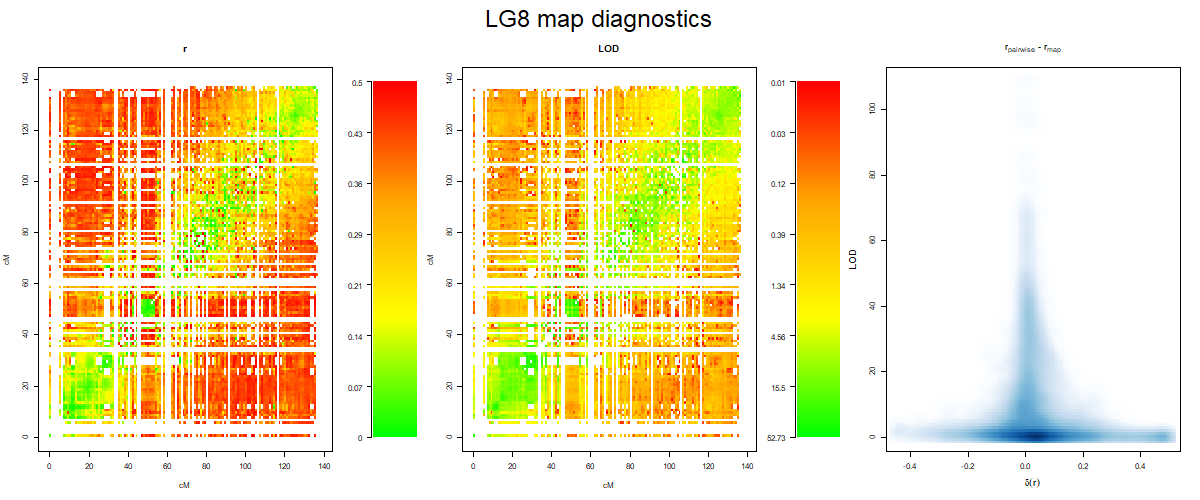


**QC (quality control) plots for each of the 8 linkage groups on the leek integrated genetic map. The first plot shows for each pair of markers their pairwise recombination frequency plotted against their map positions. The second plot is similar, except that LOD values are shown in place of recombination frequencies. Marker pairs near the diagonal should be closely linked, implying a low recombination and a high LOD, both shown as green. The third plot shows for each marker pair their LOD compared to the difference between their direct recombination and the recombination expected from their distance on the map; if this difference is large the LOD should be small.**

## S9 Linkage map comparison
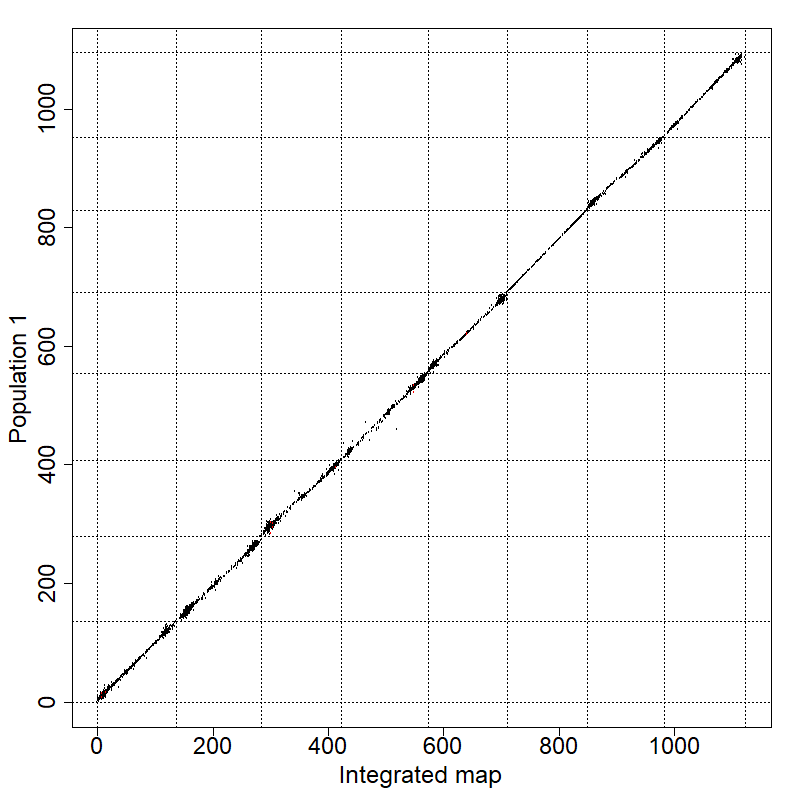


**A**

**Comparison of marker positions on the linkage maps generated from Population 1 (A) and Population 2 (B) with their positions on the integrated linkage map (x-axis). Close correspondence indicates collinearity between the population-specific and integrated maps.**


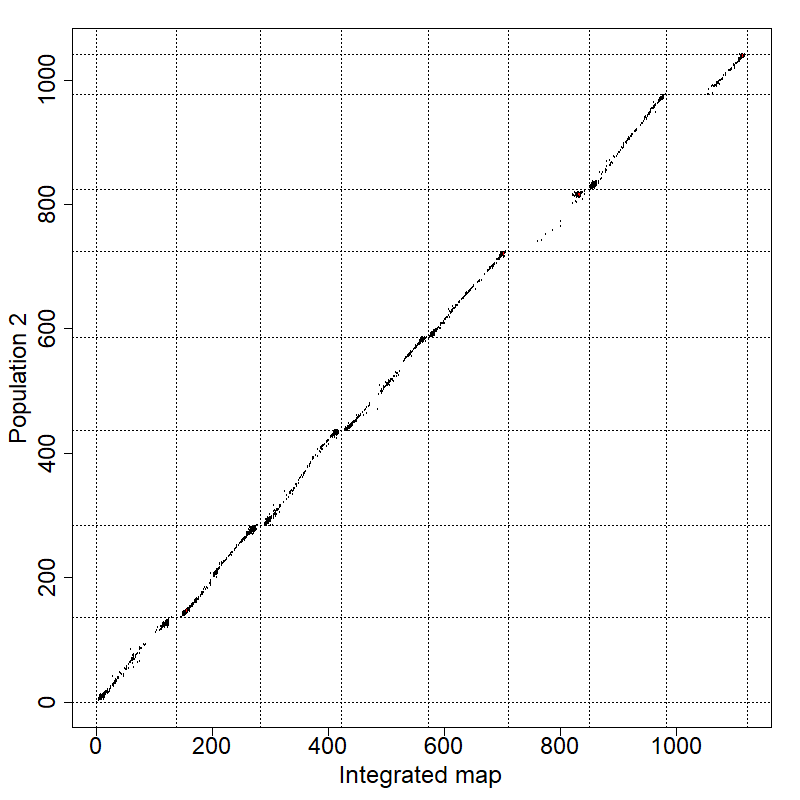


**B**

## S10 Scaffolding per linkage group
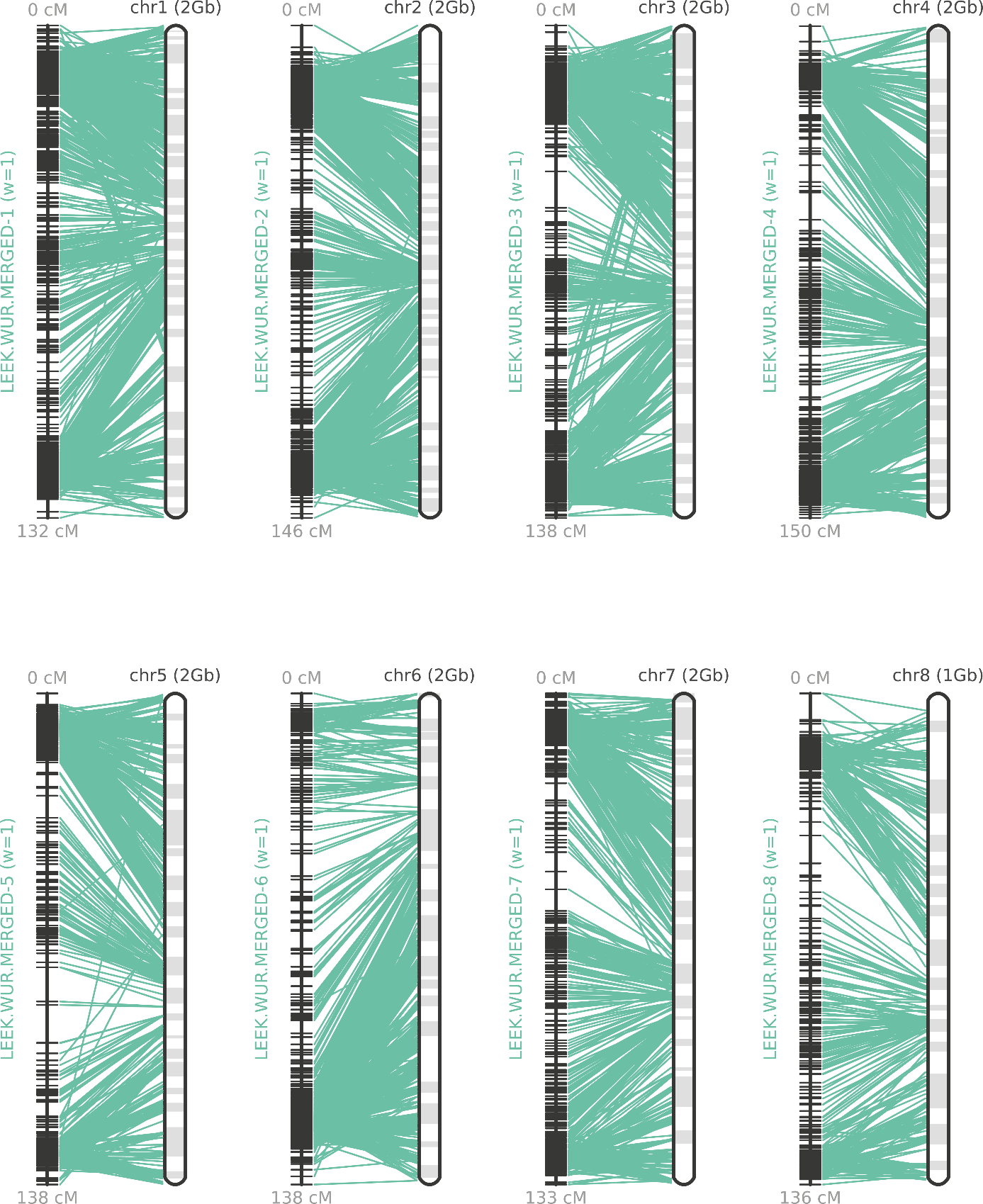


**Scaffolding output from AllMaps showing the placement of markers on physical chromosomes. For each chromosome the left part represents the linkage map and the right part the corresponding scaffolded chromosomes. Genetic marker (left) and corresponding physical marker positions (right) are shown by connector lines. Top and bottom row from left to right display chr1–chr4 and chr5–chr8, respectively.**

## S11 Marker density, rDNA annotation, centromere localization markers

**Chromosome plots for each *A. porrum* chromosome (numbered left of the karyoplot track), with tracks displayed along the chromosome length (x-axis) from top to bottom, labeled on the right-hand side:**

- **Genetic distance: Each dot represents a marker from the integrated linkage map. Left-hand and top axes show marker density along the respective dimension.**
- **Repeat density: From RepeatModeler/RepeatMasker annotation.**
- **Gene density (Helixer): Ab initio predicted genes.**
- **Gene density (Braker3): Combined ab initio and evidence-based gene predictions.**
- **Assembly contigs: Karyoplot of scaffolded de novo assembly contigs (unlabeled track).**
- **Centromeric satellite repeats: BLASTn hits for *A. cepa* AceSat02-750, AceSat01-377, AcCen1K, and *A. fistulosum* AfCen1K.**
- **rDNA sequences: Infernal hits for 5S, 5.8S, 18S, and 28S sequences.**

**Notes:**

1. **Vertical yellow lines on chromosomes 1–3 indicate image splicing due to exceeding maximum chromosome length; some distortion may be observed.**
2. **All density tracks are calculated with a bin size of 15 Mbp; smaller bin sizes are not feasible given the large chromosome sizes.**


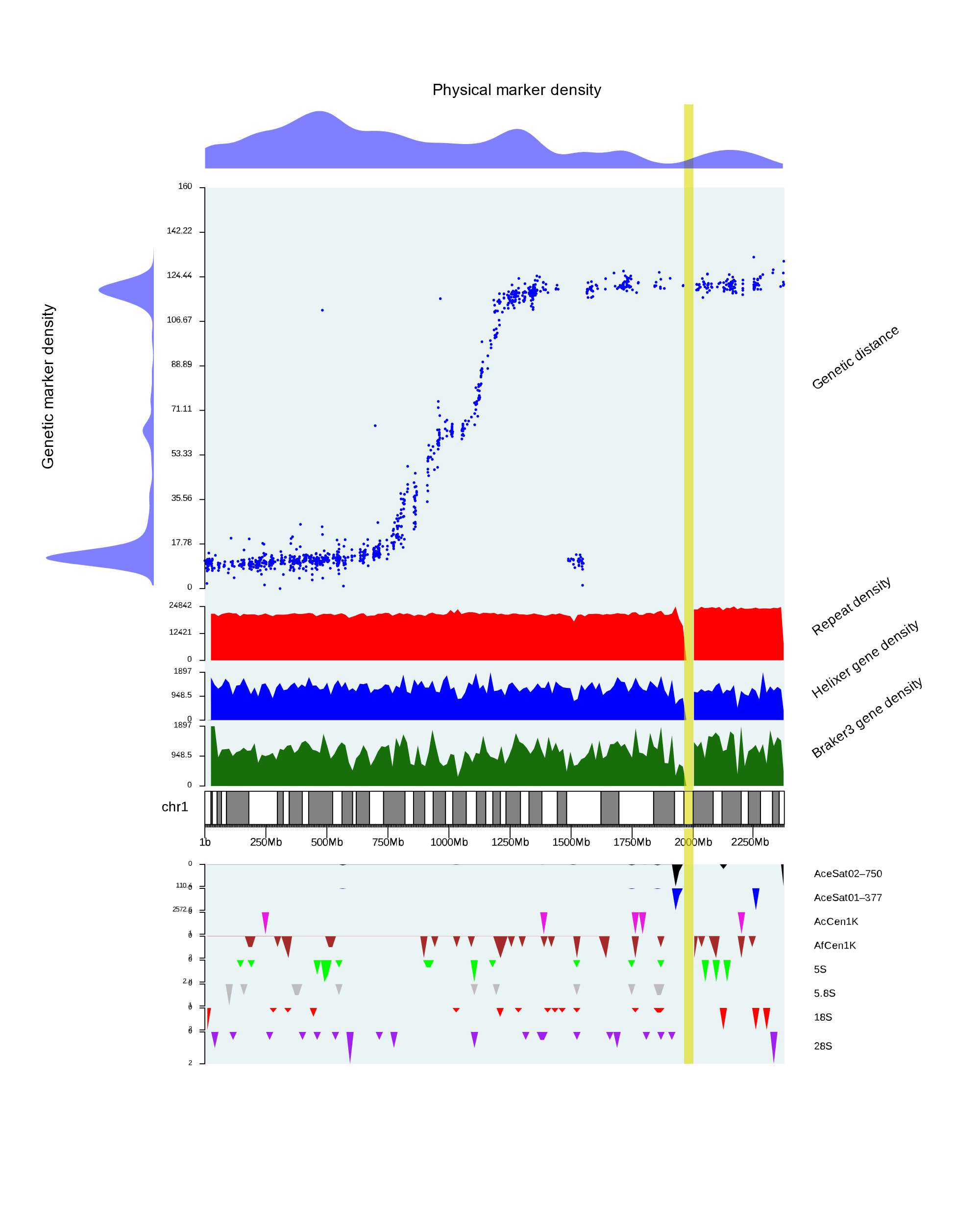


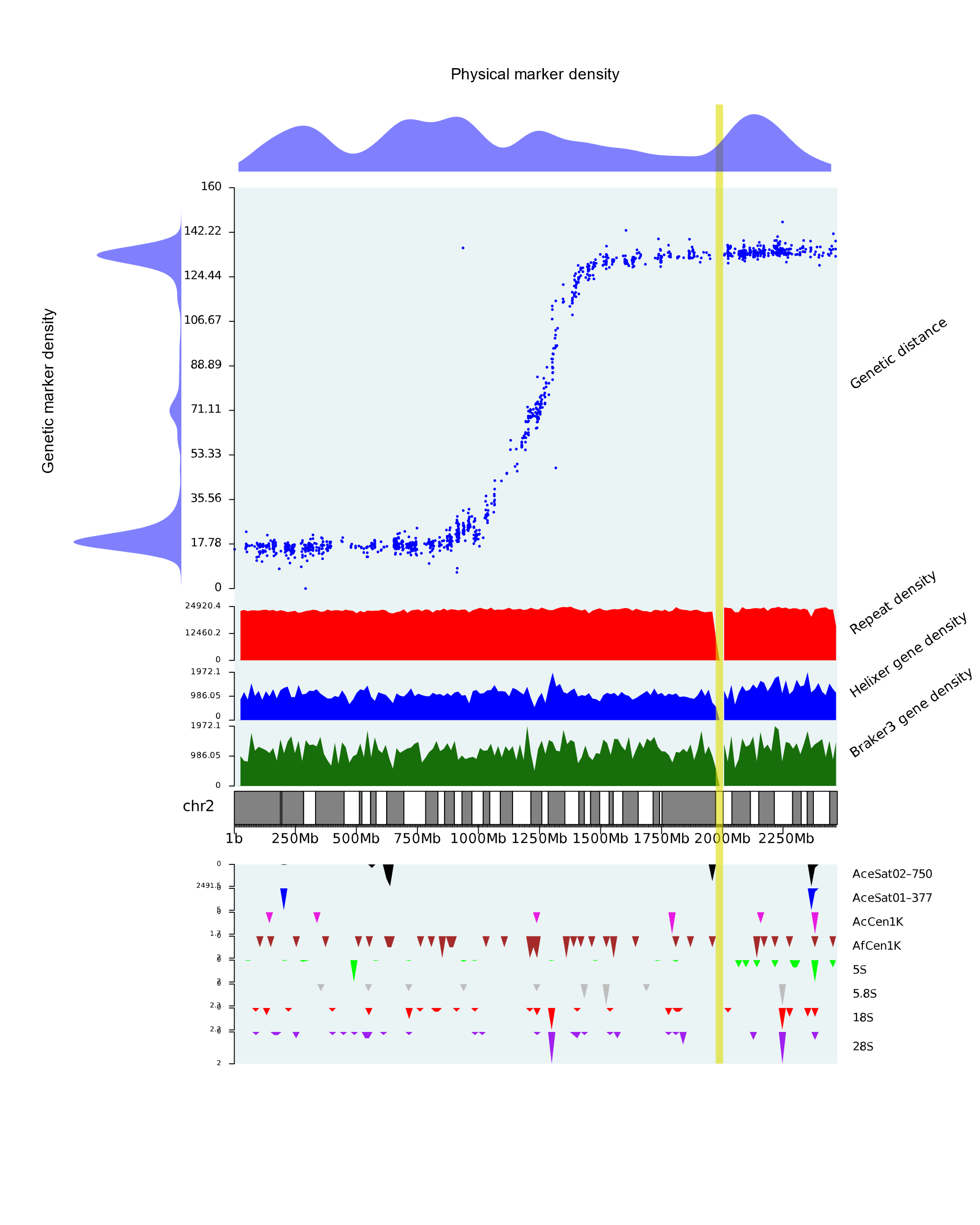


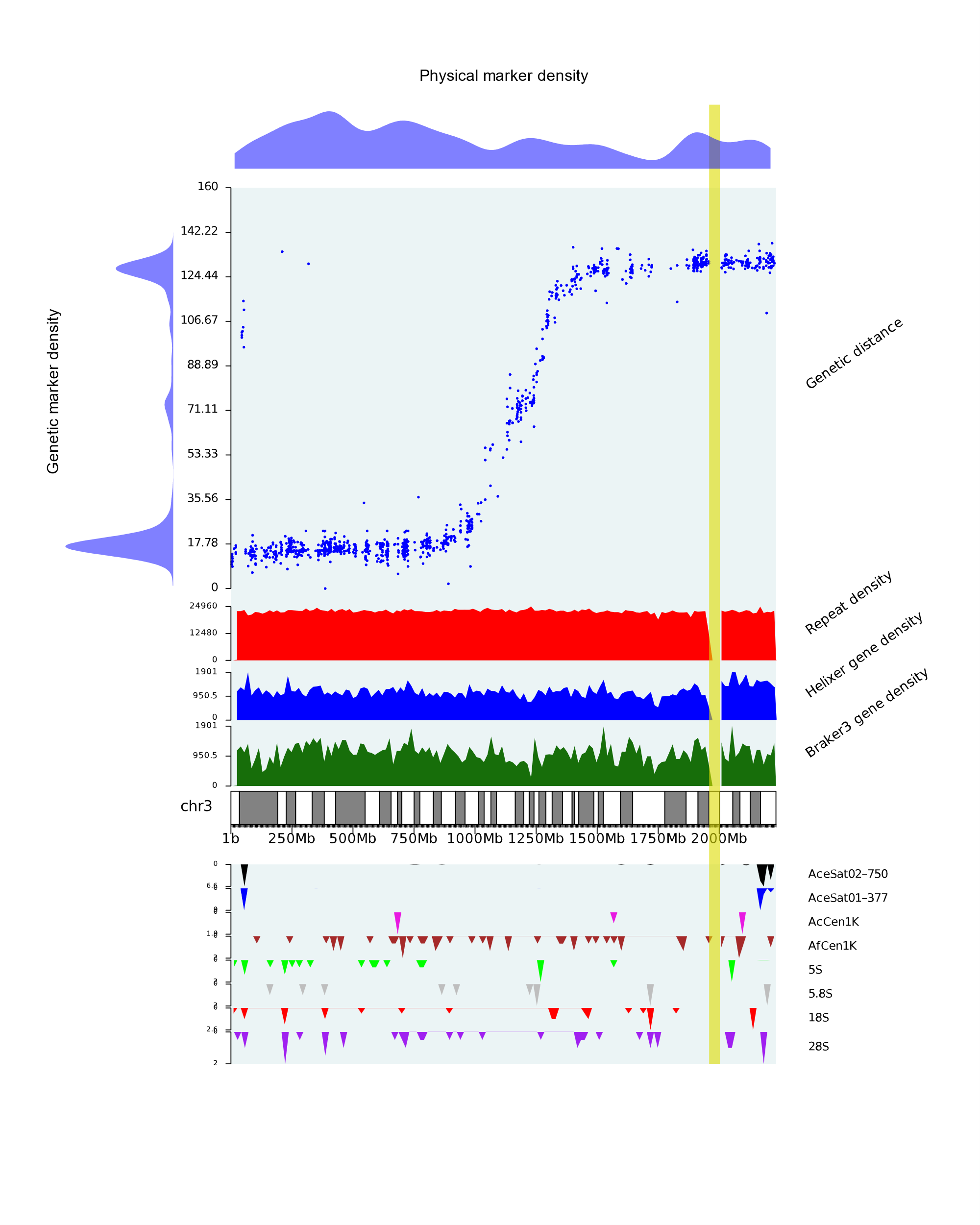


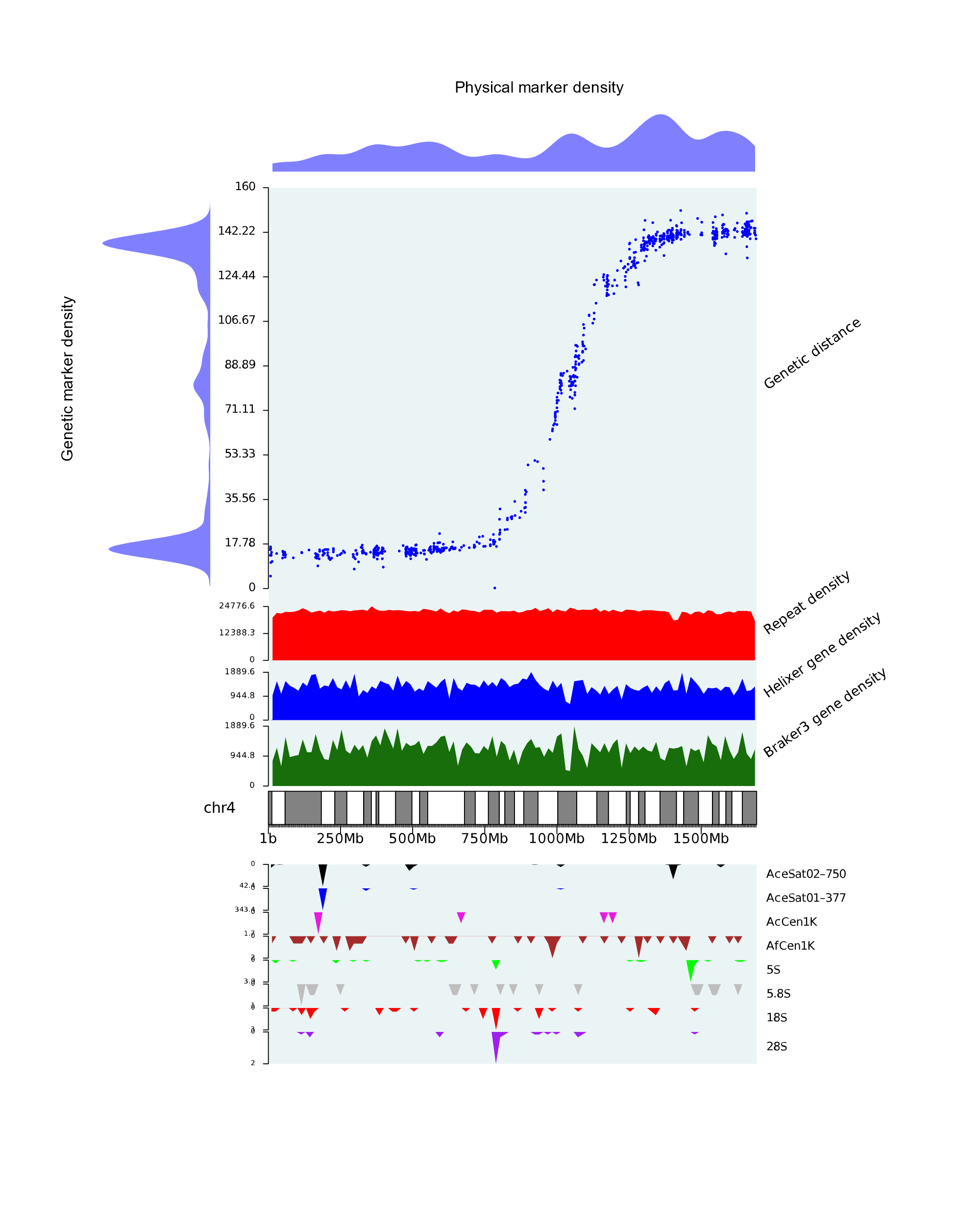


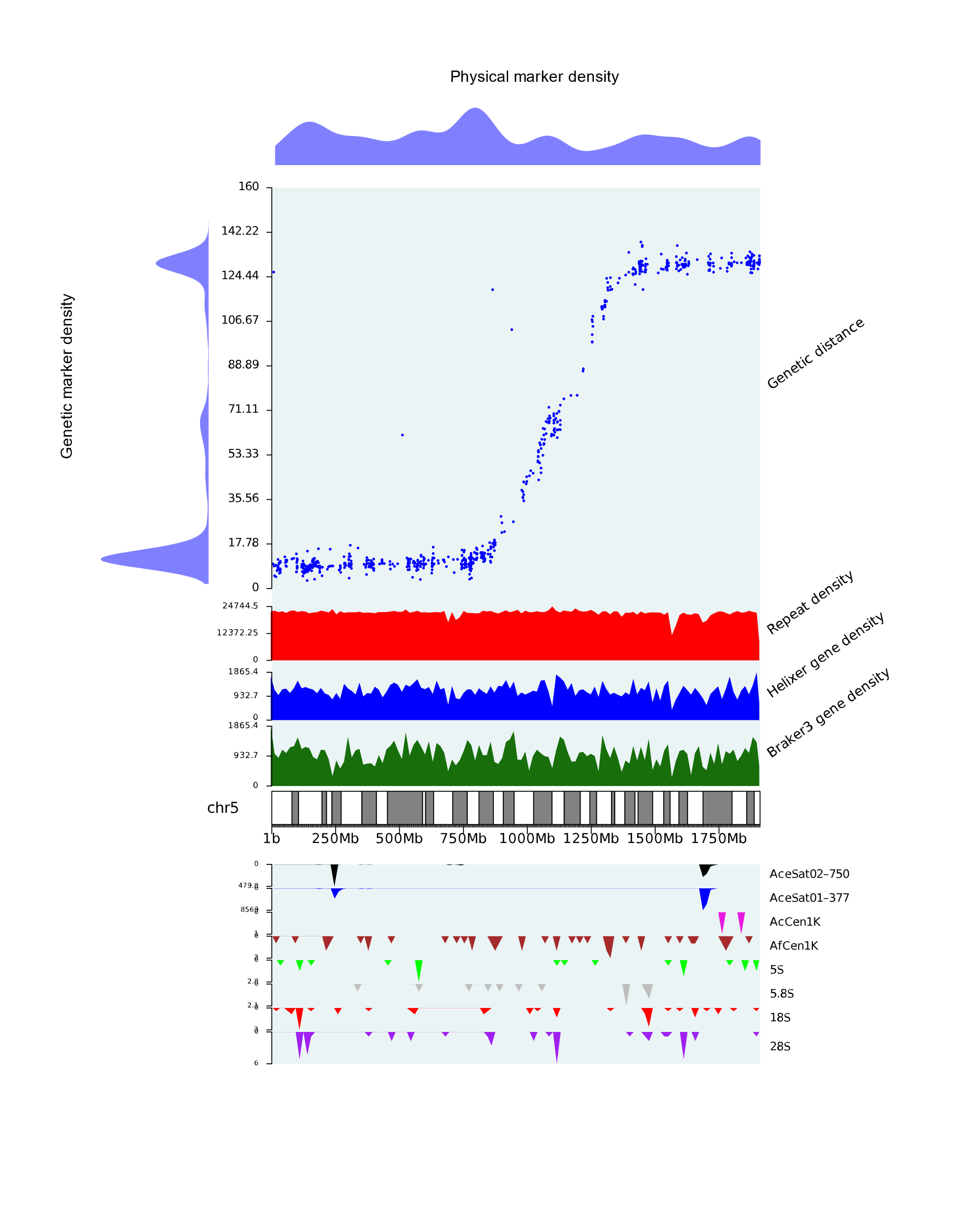


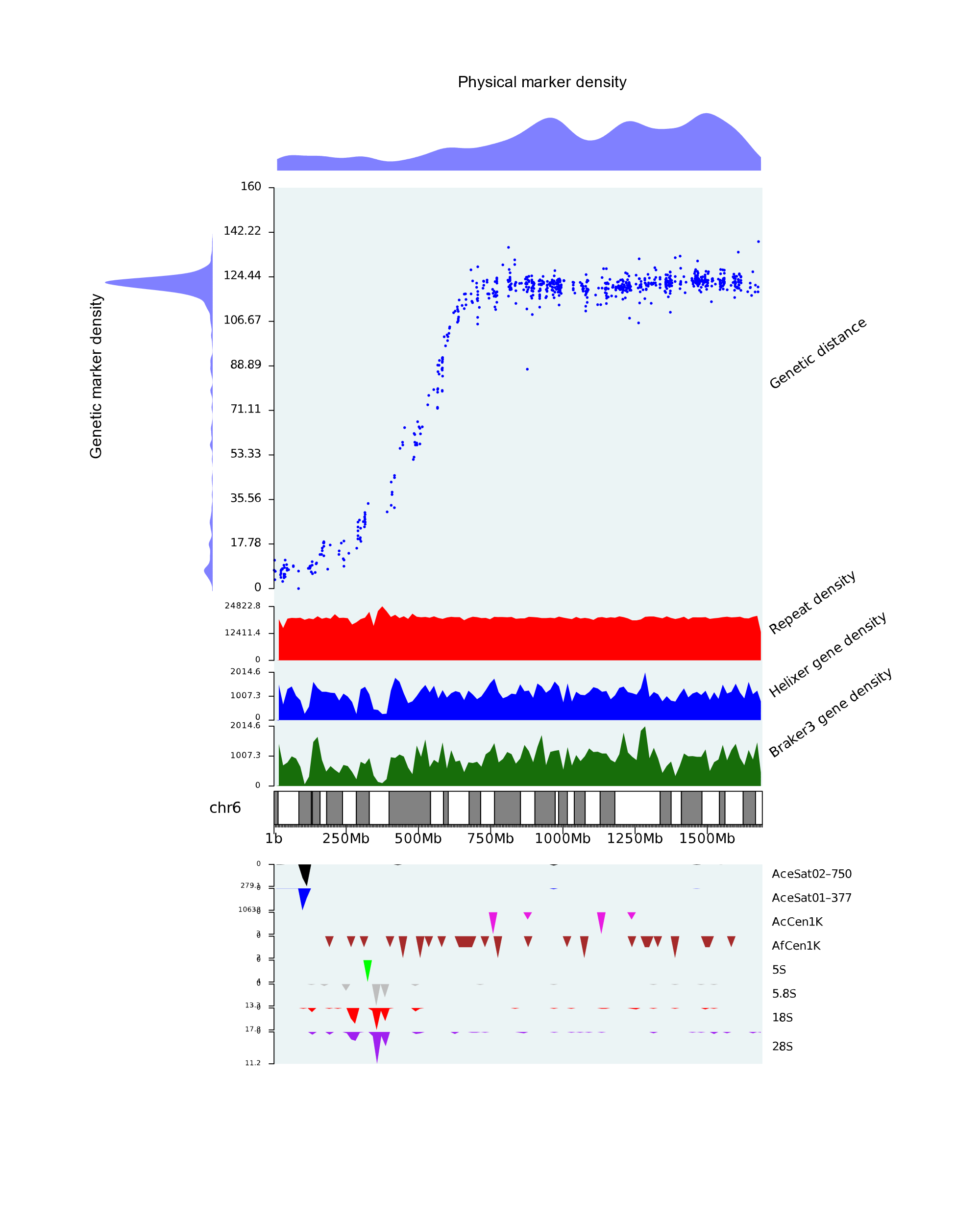


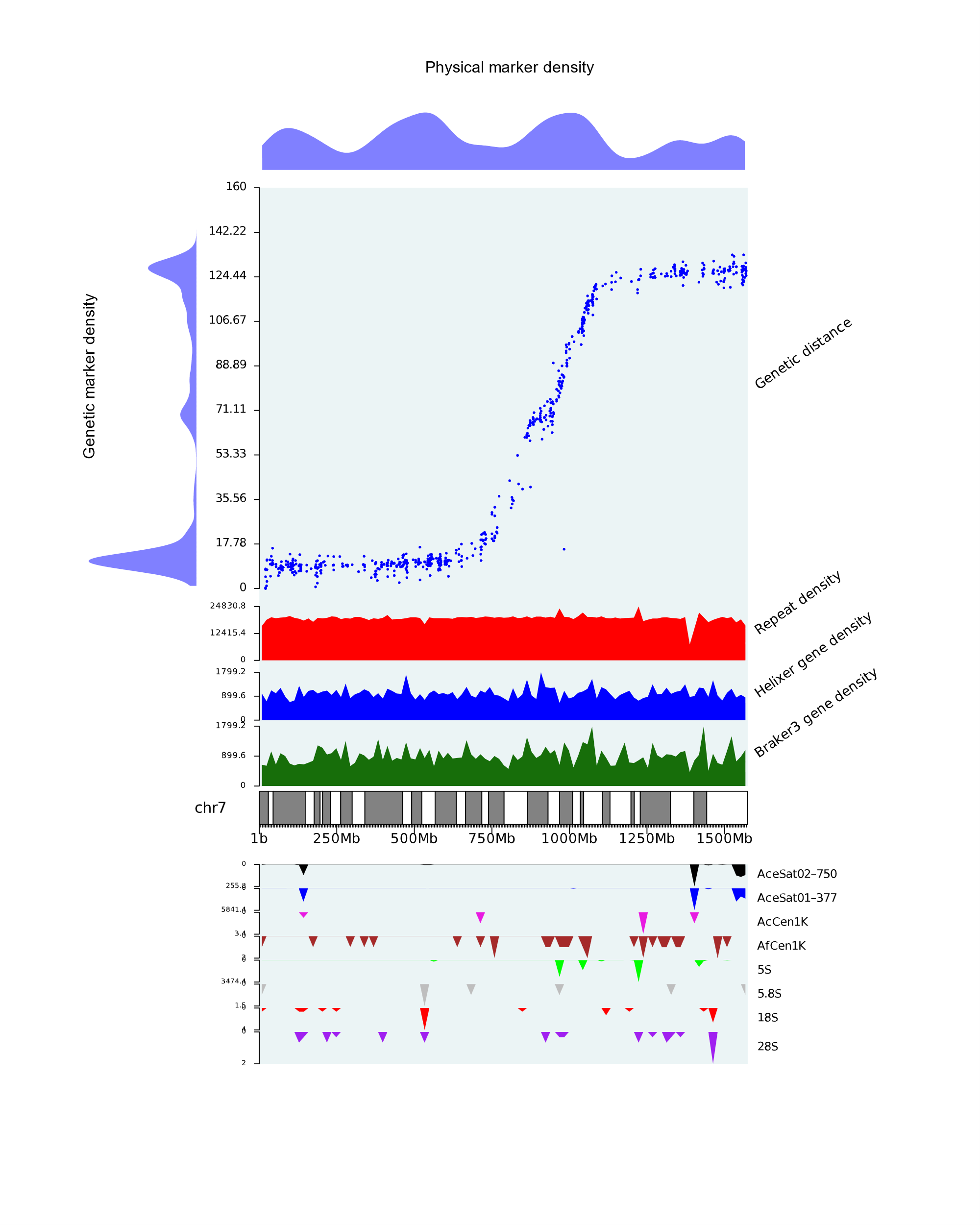


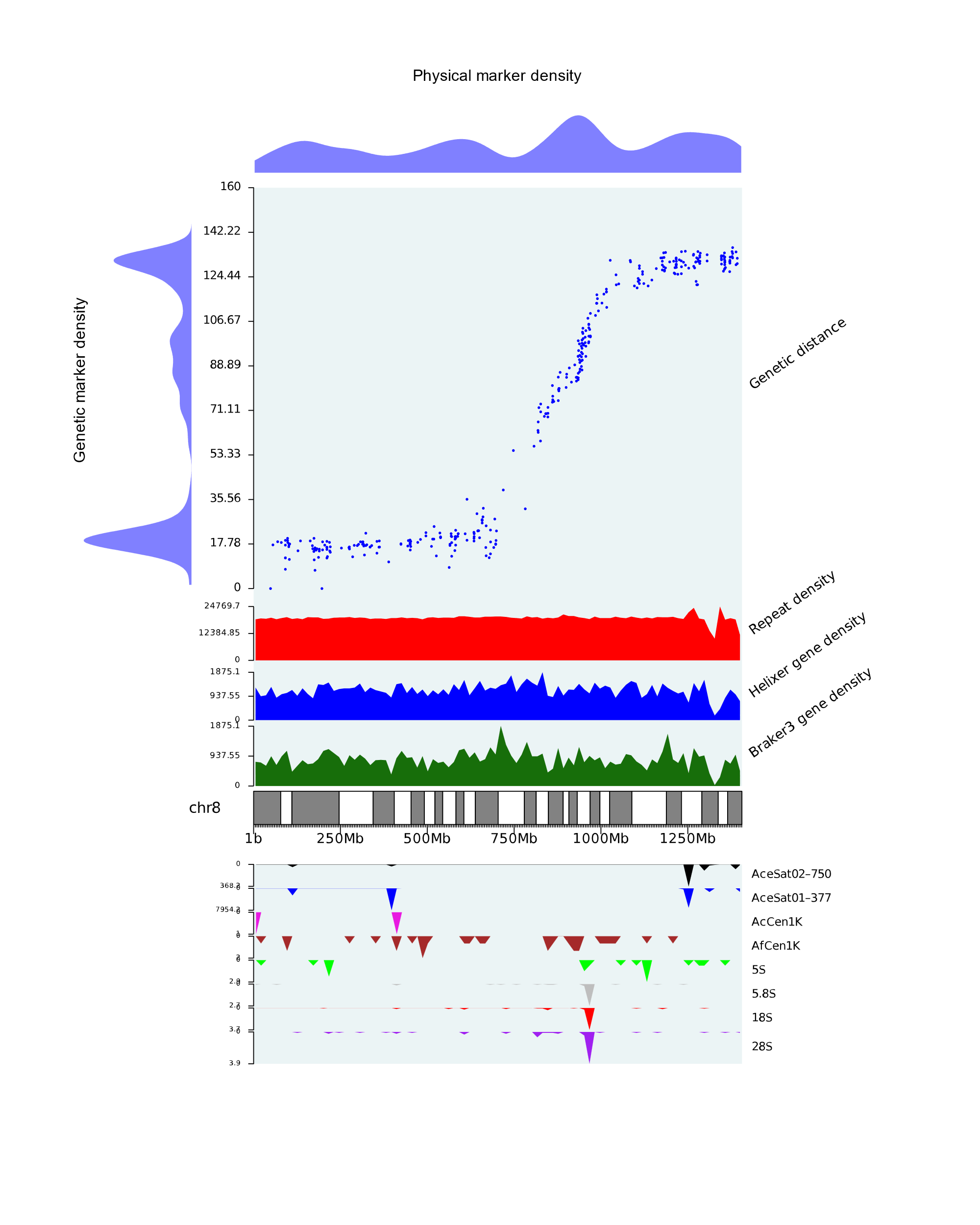


## S12 Physical marker mappings between A. porrum and A. sativum (2021), & A. cepa

**Mapping of *A. porrum* markers to the physical genomes of garlic (*A. sativum*; panel A; Sun *et al*., 2020) and onion (*A. cepa*; panel B; Hao *et al*., 2023). Each panel shows, for each chromosome, the position of markers on the *A. porrum* genome (x-axis) versus their position in the other genome (y-axis). Colors indicate the linkage group of each marker.**


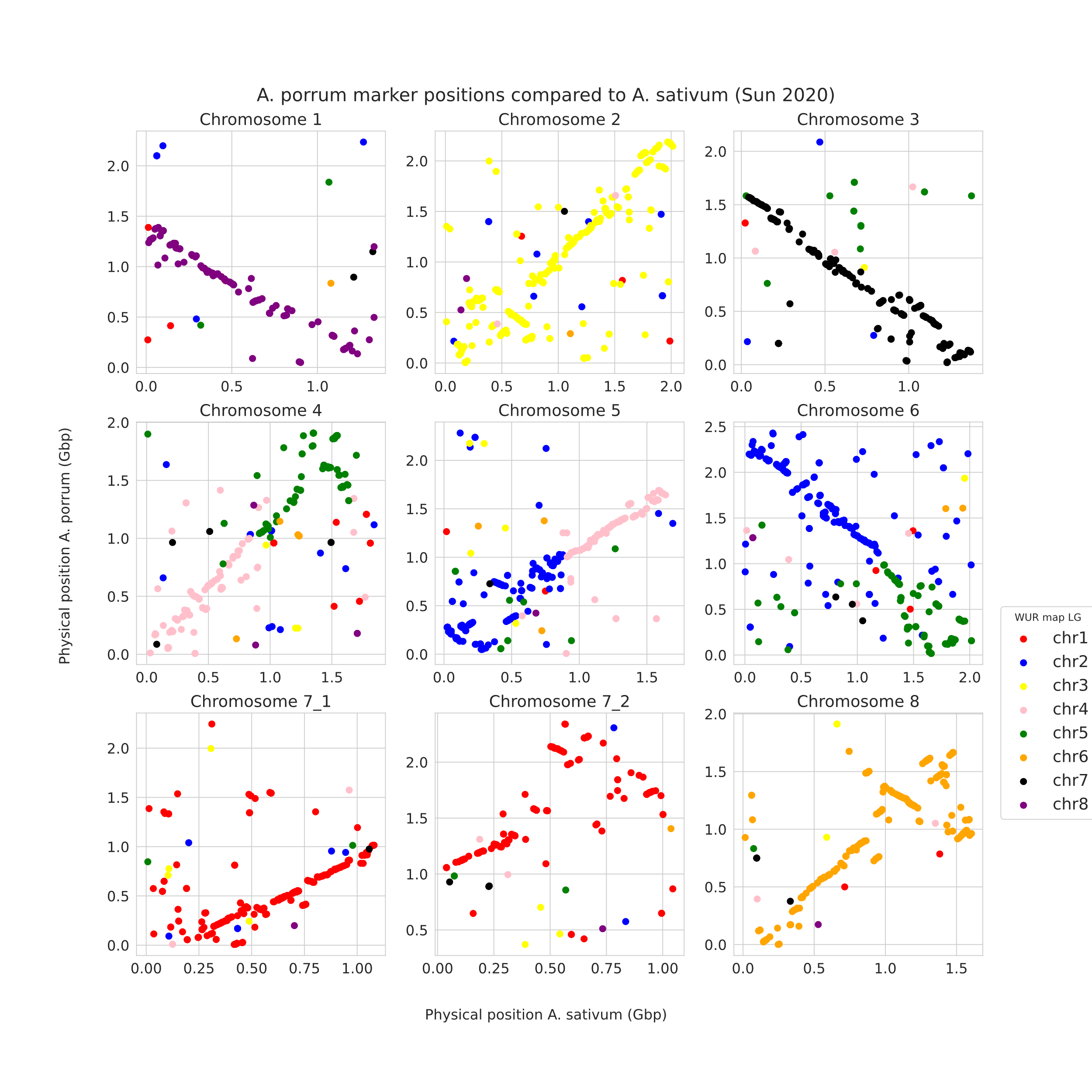


**A**

**B**


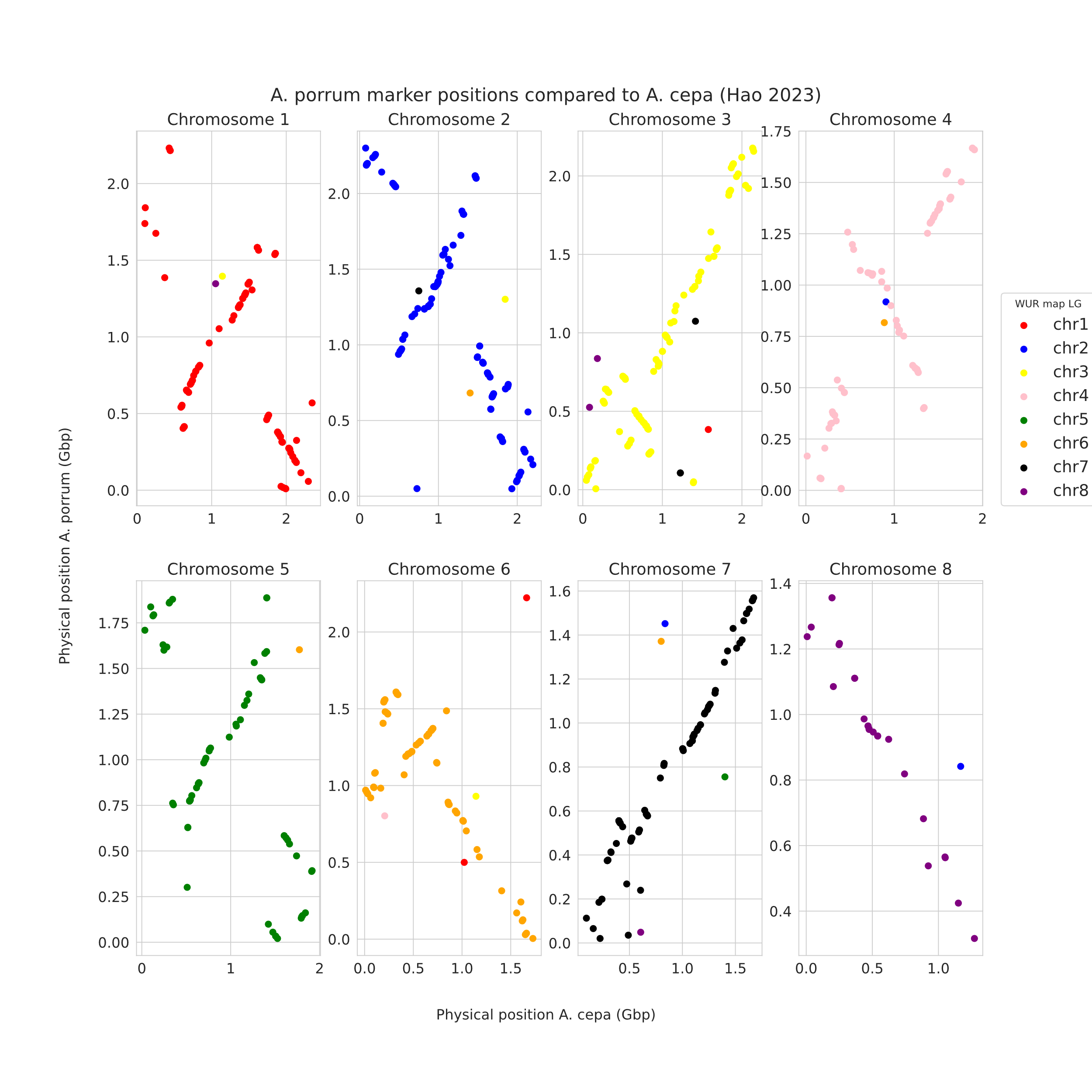

Supplement: Supplementary file 2 — Figures S1 Assembly workflow. General overview of assembly process Figures S2 Custom purging pseudocode. Describes method on how purging of primary contigs was done Figures S3 HiFi read lengths. Read length distribution of all combined HiFi reads Figures S4 HiFi mean read quality. Mean read quality distribution of all combined HiFi reads Figures S5 Genomescope2 output. K‐mer histogram and genomescope2 model fit for k = 21 (A) and k = 48 (B) Figures S6 Smudgeplot. Smudgeplot for detection of ploidy and ploidization events Figures S7 L50 plots. Plots showing the contiguity increase for each assembly iteration Figures S8 Linkage map QC. For each linkage group there are pairwise comparisons of estimated recombination frequency and observed recombination frequencies and LOD scores Figures S9 Linkage map comparison. Comparison of the separate maps to the integrated map Figures S10 Scaffolding per linkage group. Shows how each chromosome is built from contigs and the marker order from AllMaps output Figures S11 Feature density on chromosome level assembly. Visualized the feature density for repeats, genes, markers, ribosomal DNA and some satellite repeats Figures S12 Structural homology to A. sativum and A. cepa. Genome comparisons based on marker mappings from our integrated map to two different version of the garlic genome and the onion genome [file TPG2-18-e70159-s001.docx]
